# Supplementary material for: Empowering AlphaFold2 for protein conformation selective drug discovery with AlphaFold2-RAVE
Source: ArXiv. 2024 Jul 4:arXiv:2404.07102v3. Originally published 2024 Apr 10. Preprint. [Version 3] (PMC11042445)
Supplement: Supplement 1 [file NIHPP2404.07102v3-supplement-1.pdf]

arXiv:2404.07102v3 [physics.bio-ph] 4 Jul 2024

Supplementary Information: Empowering AlphaFold2 for protein conformation selective drug discovery with AlphaFold2-RAVE

Xinyu Gu,[†,¶] Akashnathan Aranganathan,[†,§] and Pratyush Tiwary[*,†,‡,¶]

†*Institute for Physical Science and Technology, University of Maryland, College Park, Maryland 20742, USA*

‡*Department of Chemistry and Biochemistry, University of Maryland, College Park 20742, USA*

¶*University of Maryland Institute for Health Computing, Bethesda, United States*

§*Biophysics Program, University of Maryland, College Park 20742, USA*

E-mail: ptiwary@umd.edu

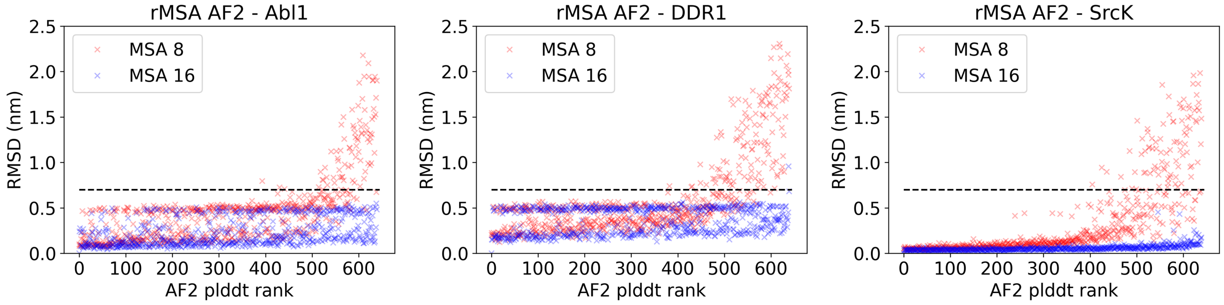

Figure S1: The AF2 pLDDT rank is plotted against the CA RMSDs from the AF2 structure (the one with the highest pLDDT) for each structure in the rMSA AF2 ensemble for Abl1, DDR1 or Src kinase. A RMSD cutoff of 7 Å (dashed black line) is applied to filter out unphysical structures with large RMSD from the native structure. Each rMSA AF2 ensemble is consist of 1280 structures, 640 for MSAs of depth 8:16 (red) and 16:32 (blue), separately.

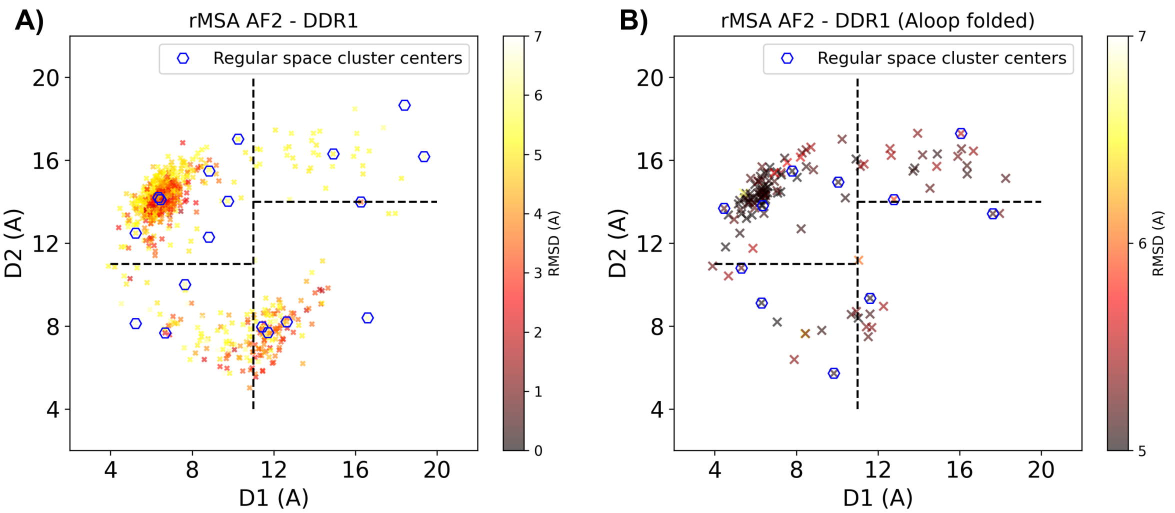

Figure S2: A) the rMSA AF2 ensemble for DDR1 is projected in the Dunbrack space. Sample points are color-coded based on the CA RMSD from the AF2 structure with the highest pLDDT. Regular space cluster centers are marked by blue hexagons. For each DFG-type (in, inter or out), top two cluster centers with the lowest CA RMSD are selected as AF2RAVE initial structures. B) To take account of the underrepresented A-loop folded configurations, an extra regular space clustering is conducted only for the A-loop folded structures in the rMSA AF2 ensemble. The color code, notation and the way to select initial structures are the same as plot A. Combining AF2RAVE initial structures from both plot A&B, there are 12 initial structures in total.

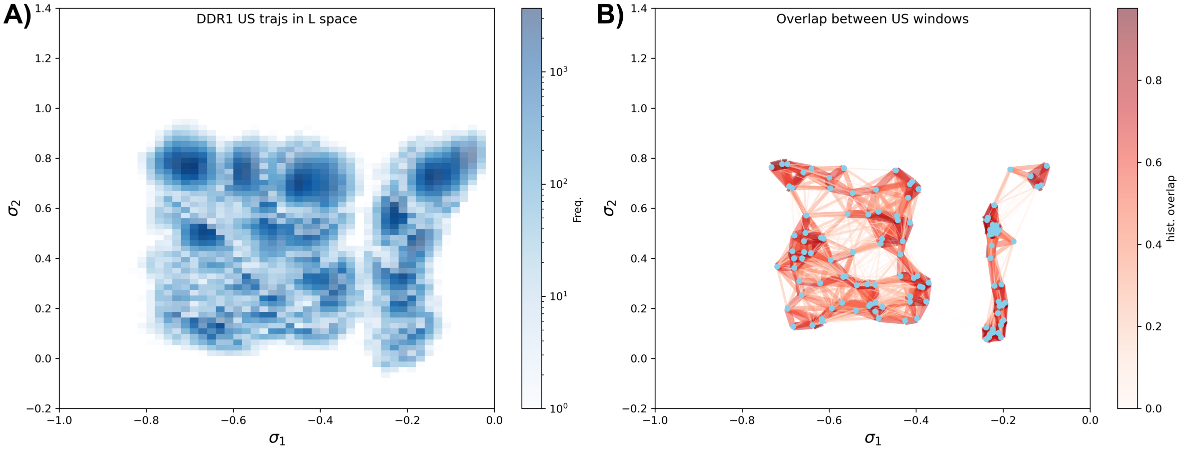

Figure S3: A) Distributions from different umbrella sampling windows in the latent space. B) The distribution overlap graph for all the umbrella sampling windows. The mean value of each distribution is shown as blue dots. Each distribution's 2D histogram is flattened into 1D vectors, and the cosine similarity between two distributions is then indicated by the width and color of the edge connecting the respective dots. Windows from the A-loop folded region are not overlapped well with the windows from the A-loop extended region, while windows inside the A-loop folded region (the left part of the graph) are well connected and are used for the local PMF calculation in Figure 4D.

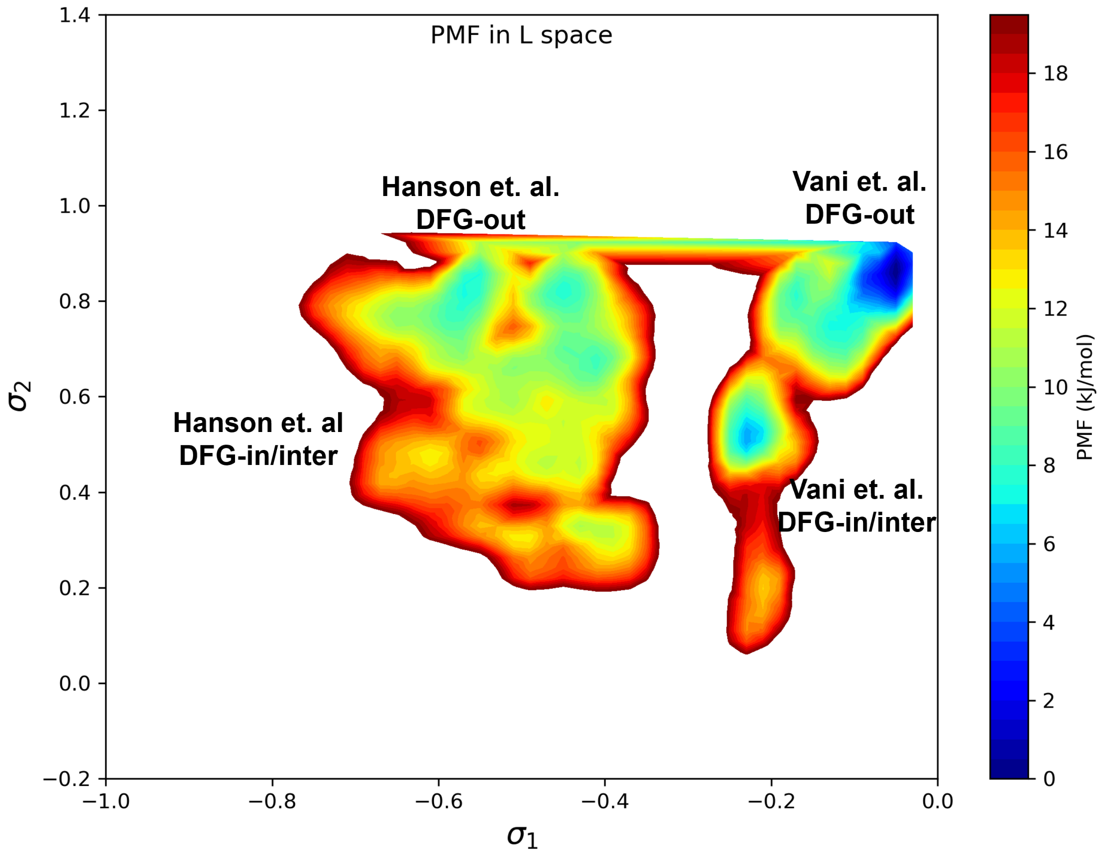

Figure S4: DDR1 PMF calculated with all the umbrella sampling windows. Hanson et al.[1] found the A-loop folded DFG-out state to be more stable than the A-loop folded DFG-in/inter state for DDR1; Vani et al.[2] reported that the A-loop extended DFG-out state is more stable than the A-loop extended DFG-in/inter state for DDR1. Although our umbrella sampling setup is not sufficient to sample the A-loop movement, the observed relative stability corresponds with the findings of Hanson et al. and Vani et al.

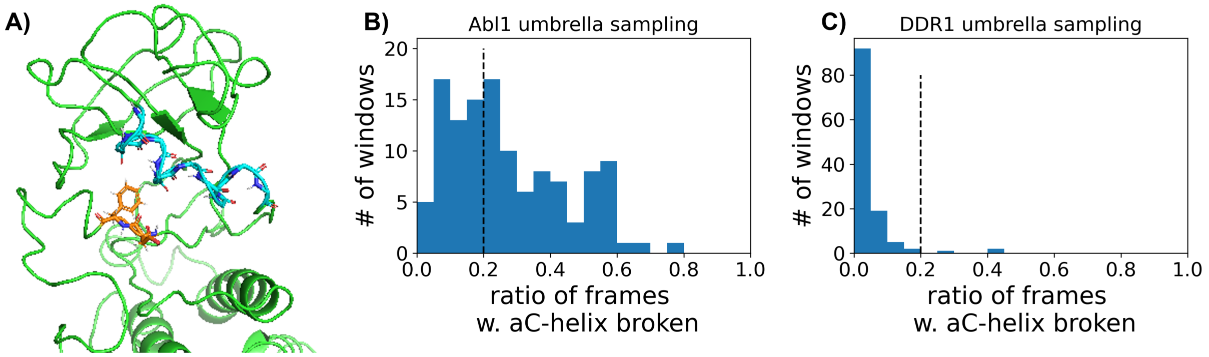

Figure S5: A) one representative frame with αC helix broken in Abl1 umbrella sampling trajectories. The backbone of the αC helix is shown with cyan sticks, while the DFG motif is shown as orange sticks. B) or C) The distribution of the ratios of frames with αC helix broken in each umbrella sampling window for Abl1 or DDR1.

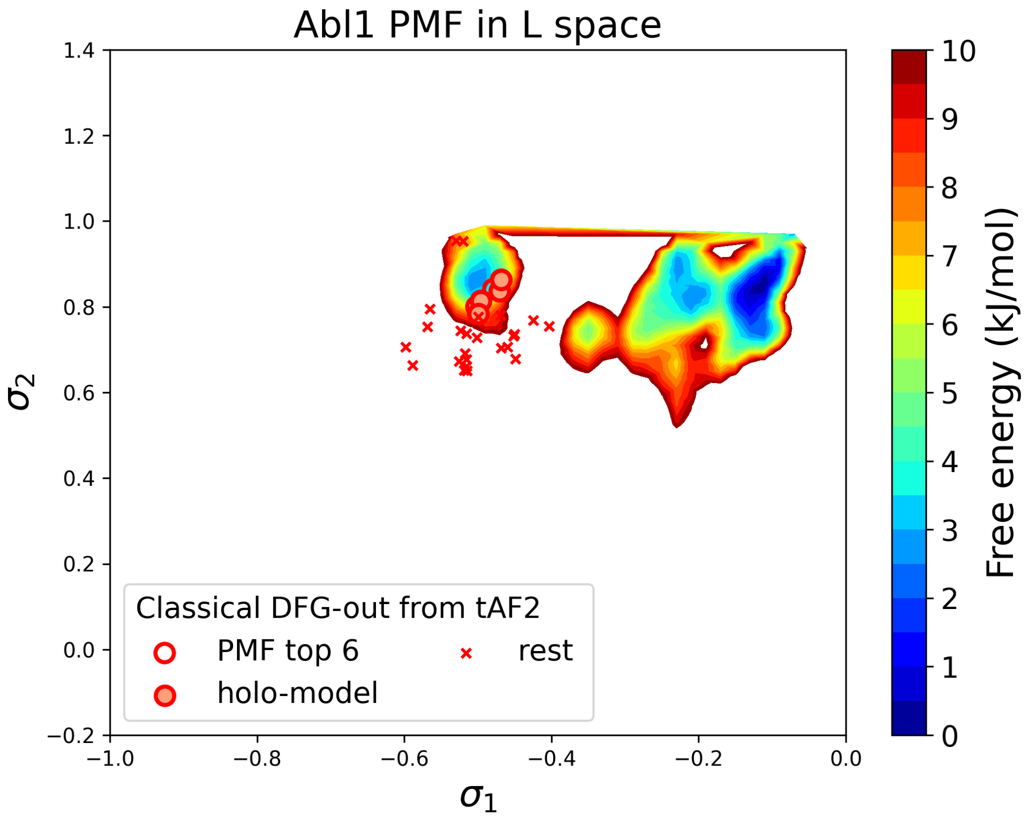

Figure S6: Abl1 PMF calculated from umbrella sampling after discarding windows with αC helix broken. The four holo-like structures ("holo-models") are enriched to the top six based on PMF values.

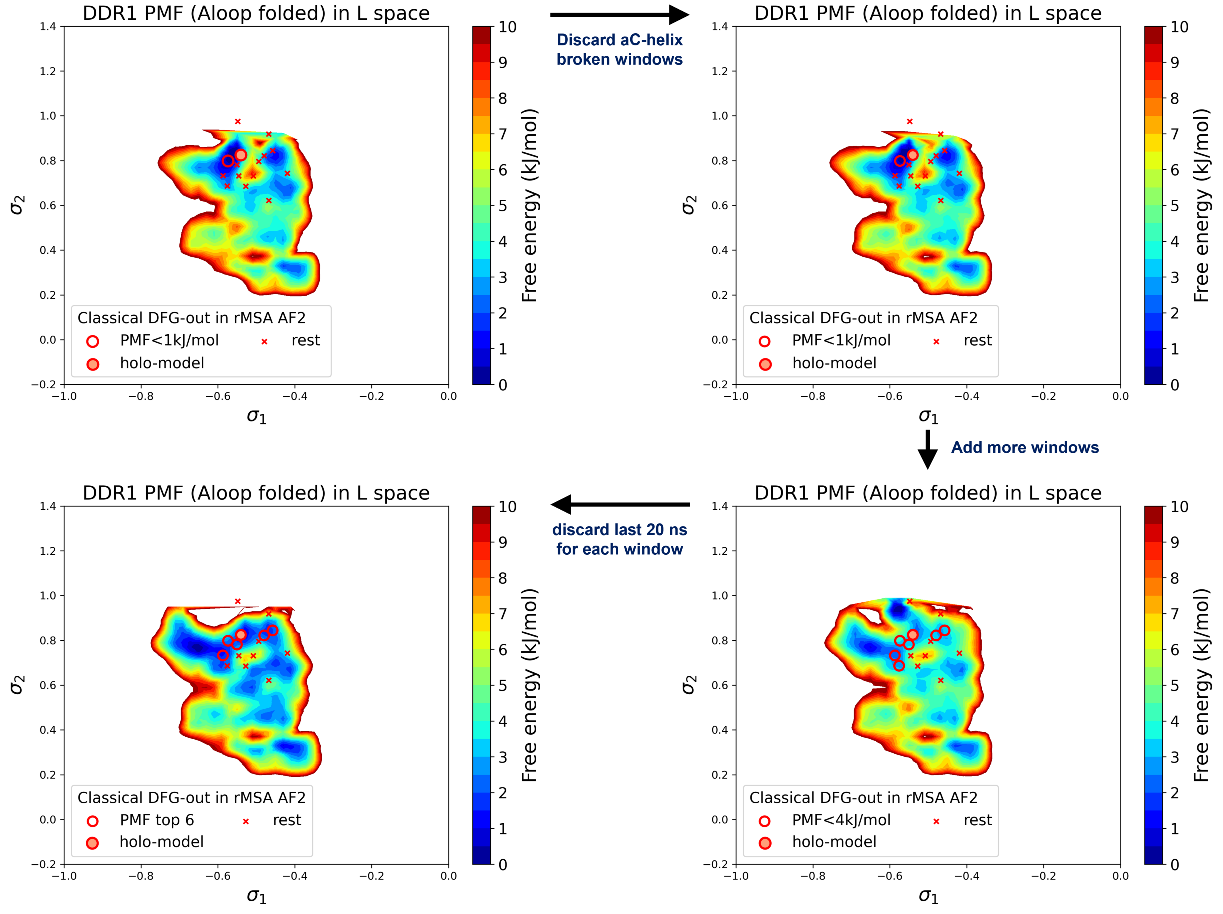

Figure S7: PMF values and Boltzmann ranks of candidate structures fluctuate with the selection of the umbrella sampling windows and the simulation length of umbrella sampling trajectories, demonstrated with the DDR1 system.

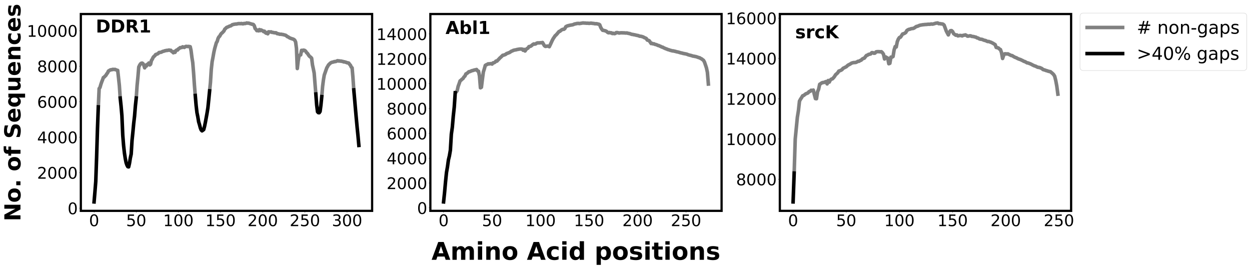

Figure S8: The plot illustrates the number of gaps in the multiple sequence alignment (MSA) generated by mmseq2 (using Colabfold[3]) for different kinases. The non-gap count describes the coverage of each position in the MSA. The presence of residue positions with gap counts higher than 40 per cent of the total sequence in DDR1 implies that it has fewer conserved regions than abl1 kinase and src kinase. This characteristic of DDR1 MSA enables the rMSA AF2 protocol to generate multiple conformations for DDR1, including the classical DFG-out conformation, by initializing it at various states. However, the highly conserved nature of abl1 and src makes it challenging for the rMSA AF2 to initialize at a state that can lead to a classical DFGout conformation. Therefore, we used the AlphaFold template protocol to overcome this initialization issue with rMSA AF2.

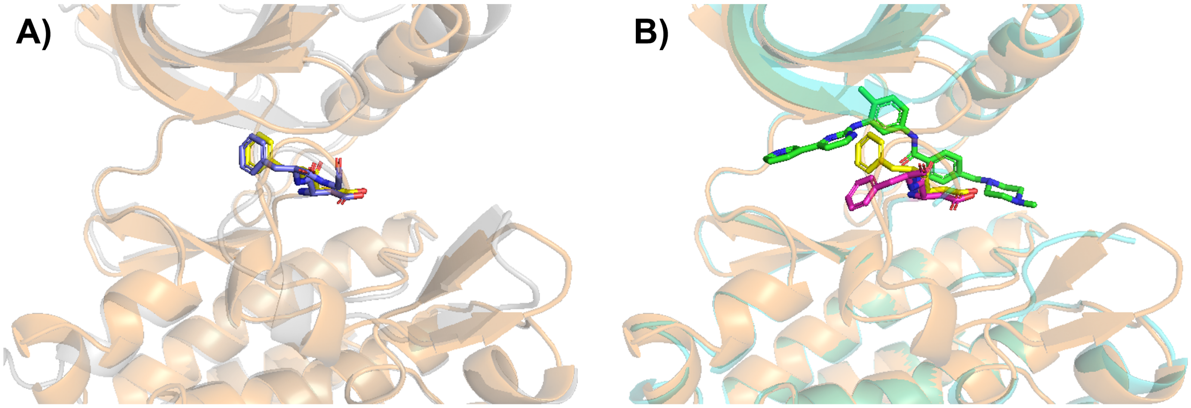

Figure S9: A) the AF2-template structure for Src kinase is superimposed with its template structure (classical DFG-out in DDR1 rMSA AF2 ensemble, "holo-model"). The tAF2 structure of Src is shown as light-orange cartoon (protein) and yellow sticks (DFG motif), while DDR1 template is shown as light-gray cartoon (protein) and blue sticks (DFG motif). B) the AF2-template structure for Src kinase is again superimposed with Src/imatinib co-crystallized structure (PDB 2OIQ). Crystal structure is shown as light-cyan cartoon (protein), green sticks (ligand) and magenta sticks (DFG motif).

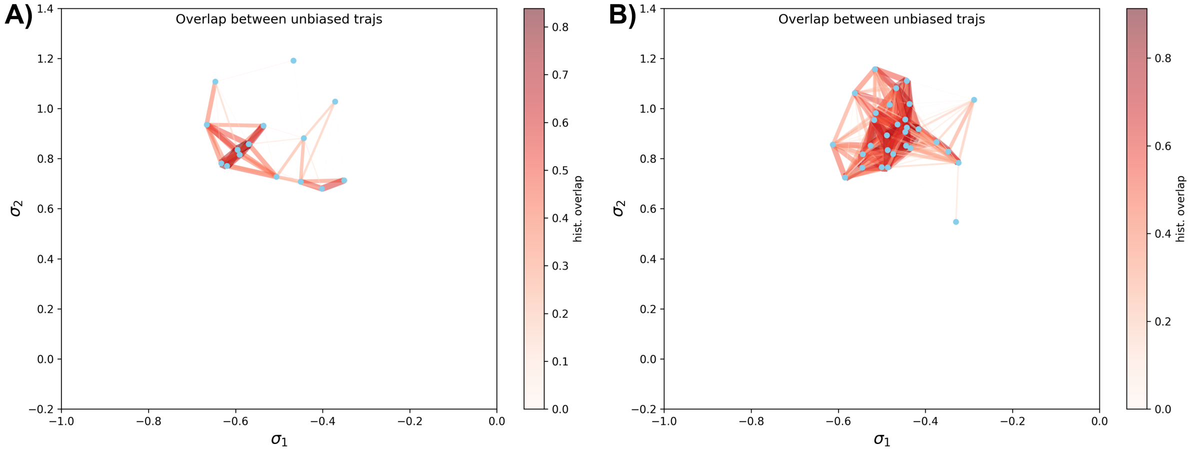

Figure S10: A) The distribution overlap graph for all the unbiased MD trajectories starting from 15 classical DFG-out structures in DDR1 rMSA AF2 ensemble. B) The distribution overlap graph for all the unbiased MD trajectories starting from 30 Abl1 tAF2 structures in classical DFG-out state. The color-code is the same as Figure S3

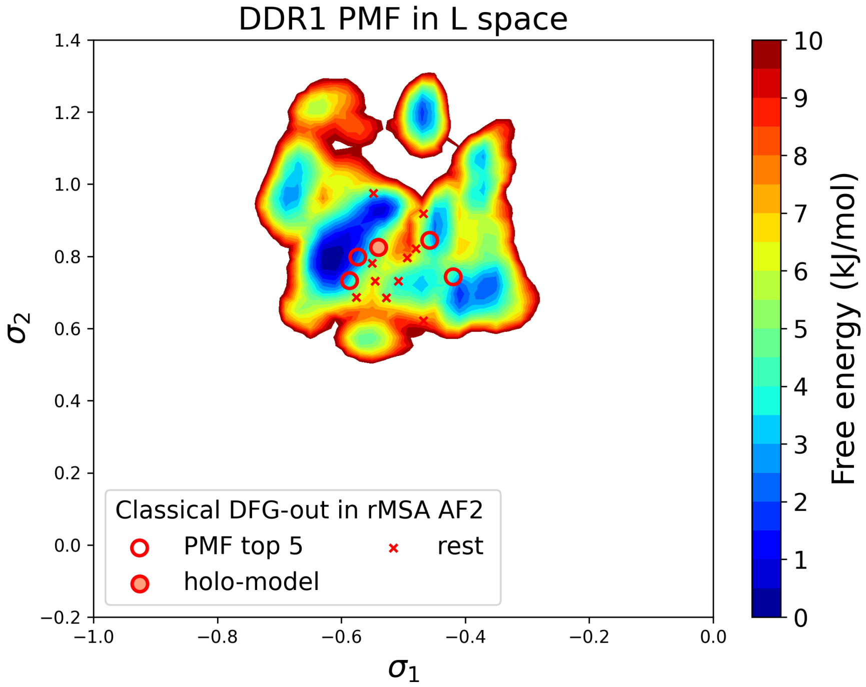

Figure S11: Free energy profile for DDR1 in the latent space, calculated from unbiased MD simulations. The 15 DDR1 classical DFG-out structures in rMSA AF2 are shown as red cross and circles (top 5 structures ranked by free energy values). The “holo-model” structure is emphasized using a red circle filled with red.

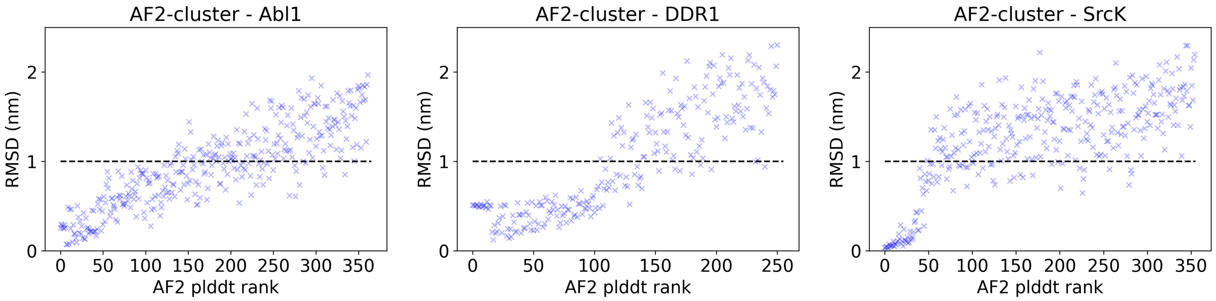

Figure S12: The AF2 pLDDT rank is plotted against the CA RMSDs from the AF2 structure for each structure in the AF2-cluster ensemble for Abl1, DDR1 or SrcK. A RMSD cutoff of 10 Å (dashed black line) is applied to filter out unphysical structures with large RMSD from the native structure. After the RMSD filter, 197 out of 362 structures remain for Abl1, 134 out of 251 structures remain for DDR1, and 93 out of 355 structures remains for SrcK.

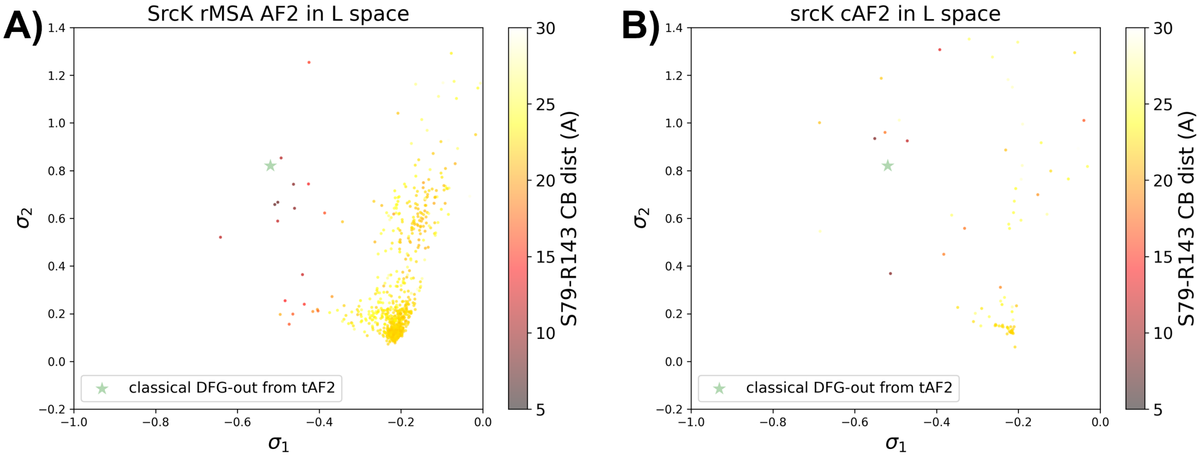

Figure S13: The projection of A) the rMSA AF2 ensemble or B) the AF2-cluster ensemble on the AF2RAVE latent space for SrcK. The classical DFG-out SrcK structure generated from AF2-template in Fig S9 is shown as the green star. The color-code shows the A-loop location.

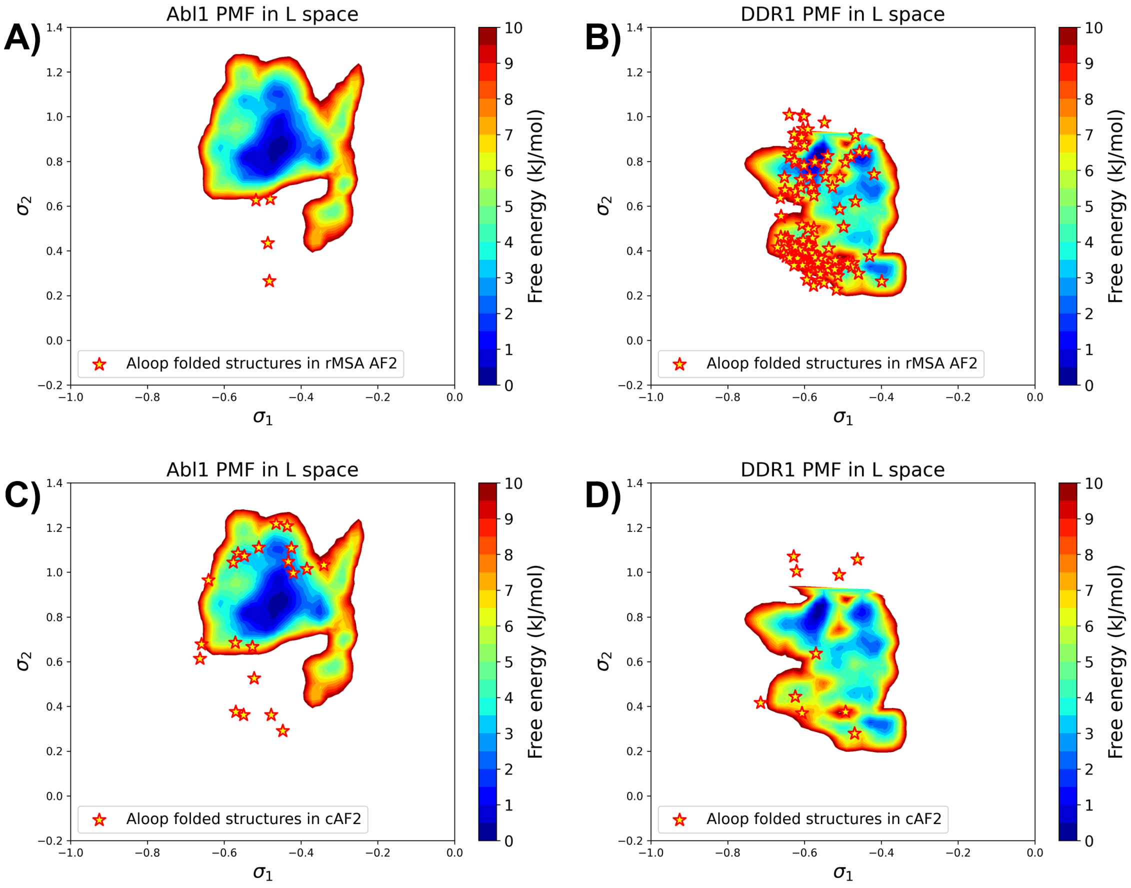

Figure S14: The projection of A-loop folded structures from the rMSA AF2 ensemble or the AF2-cluster ensemble on the AF2RAVE PMF for Abl1 or DDR1.

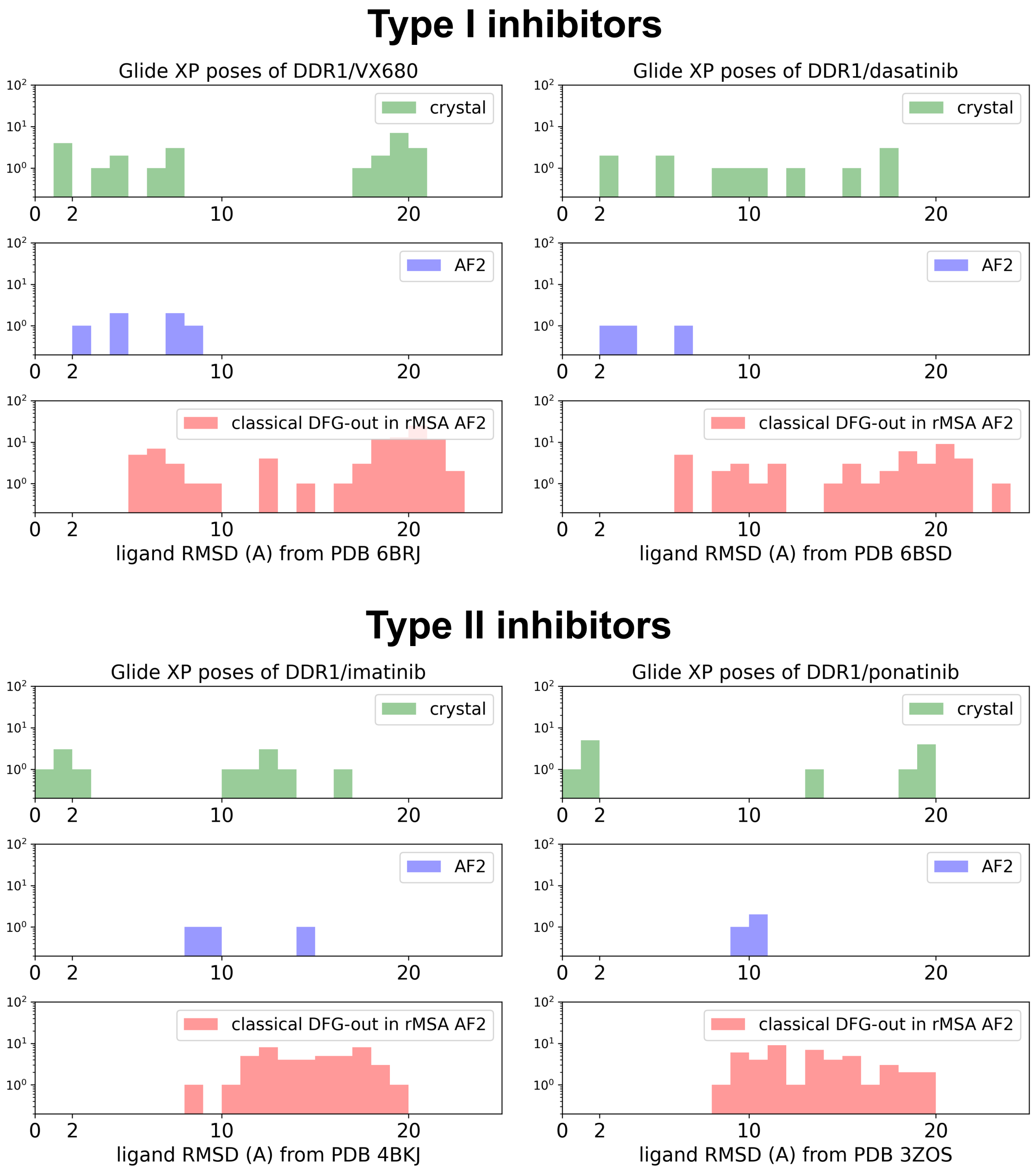

Figure S15: The distributions of ligand RMSDs for Glide XP docking poses of DDR1 and type I/type II inhibitors (upper/lower panel). Results from cross-docking against 4 crystal holo structures, docking against the AF structure, and docking against 15 classical DFG-out structure in rMSA AF2 ensemble are shown as green, blue, and red, separately.

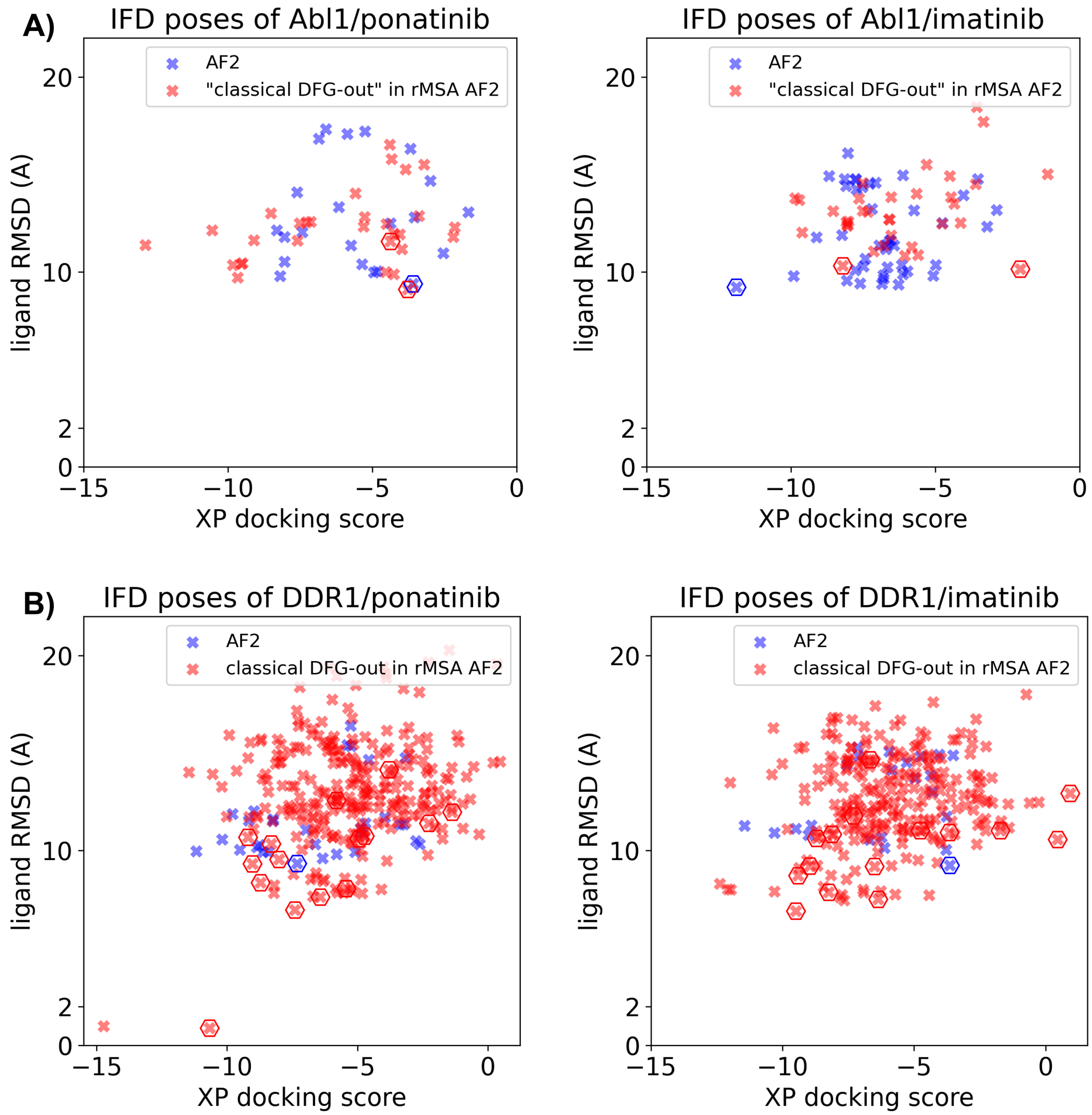

Figure S16: Ligand RMSDs are plotted against the docking scores for the IFD docking poses of type II inhibitors (ponatinib and imatinib) against AF2 structure (blue) or classical DFG-out structures in rMSA AF2 ensembles (red). A) IFD docking results for Abl1. B) IFD docking results for DDR1. The pose with the lowest ligand RMSD from each input structure is marked by hexagon.

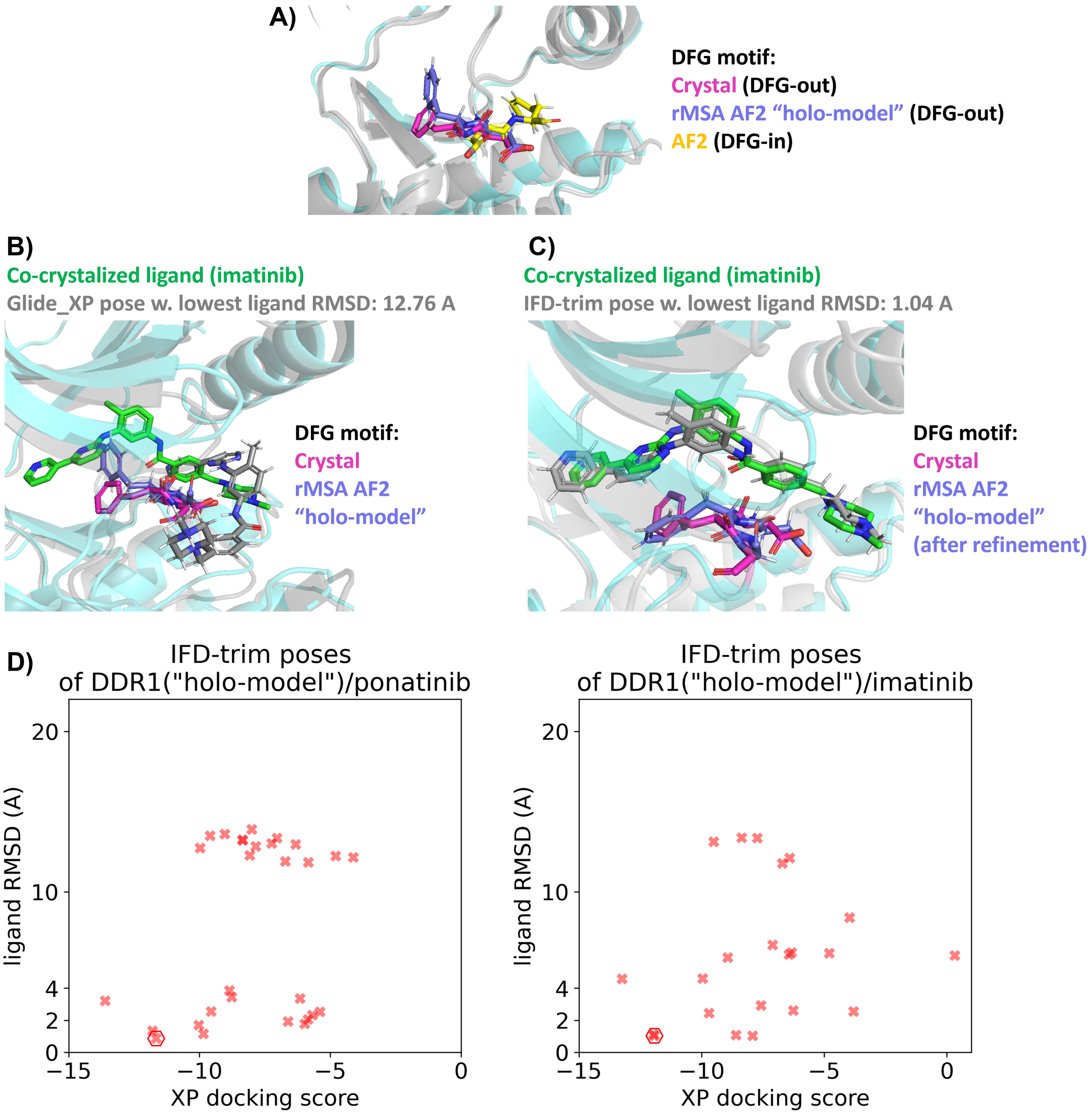

Figure S17: A) Comparison of the DFG motif for DDR1 in its co-crystalized structure with imatinib (PDB 4BKJ), its “holo-model” structure and its AF2 structure. B&C) In the “holo-model” structure, the Phe residue in the DFG-motif requires rotation to prevent steric clashes with imatinib. proteins from crystal structure are shown as cyan cartoon, while all the other proteins are shown as grey cartoon. D) Ligand RMSDs are plotted against the docking scores for the IFD-trim docking poses of type II inhibitors (ponatinib and imatinib) against the “holo-model” structure in DDR1 rMSA AF2 ensembles. The pose with the lowest ligand RMSD is marked by hexagon.

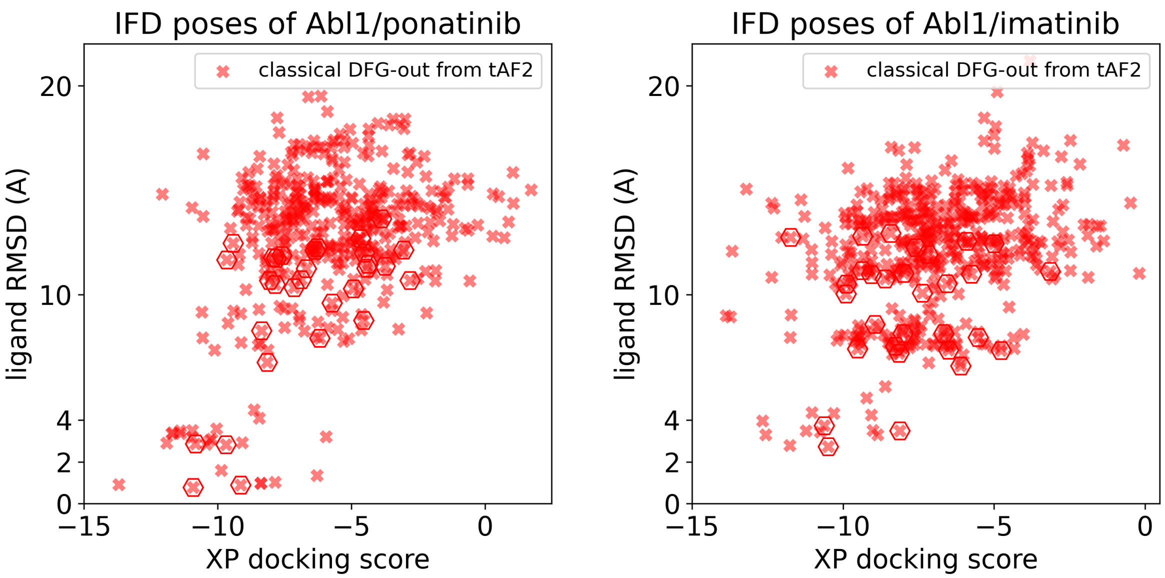

Figure S18: Ligand RMSDs are plotted against the docking scores for the IFD docking poses of type II inhibitors (ponatinib and imatinib) against Abl1 tAF2 structures. The pose with the lowest ligand RMSD from each input structure is marked by hexagon.

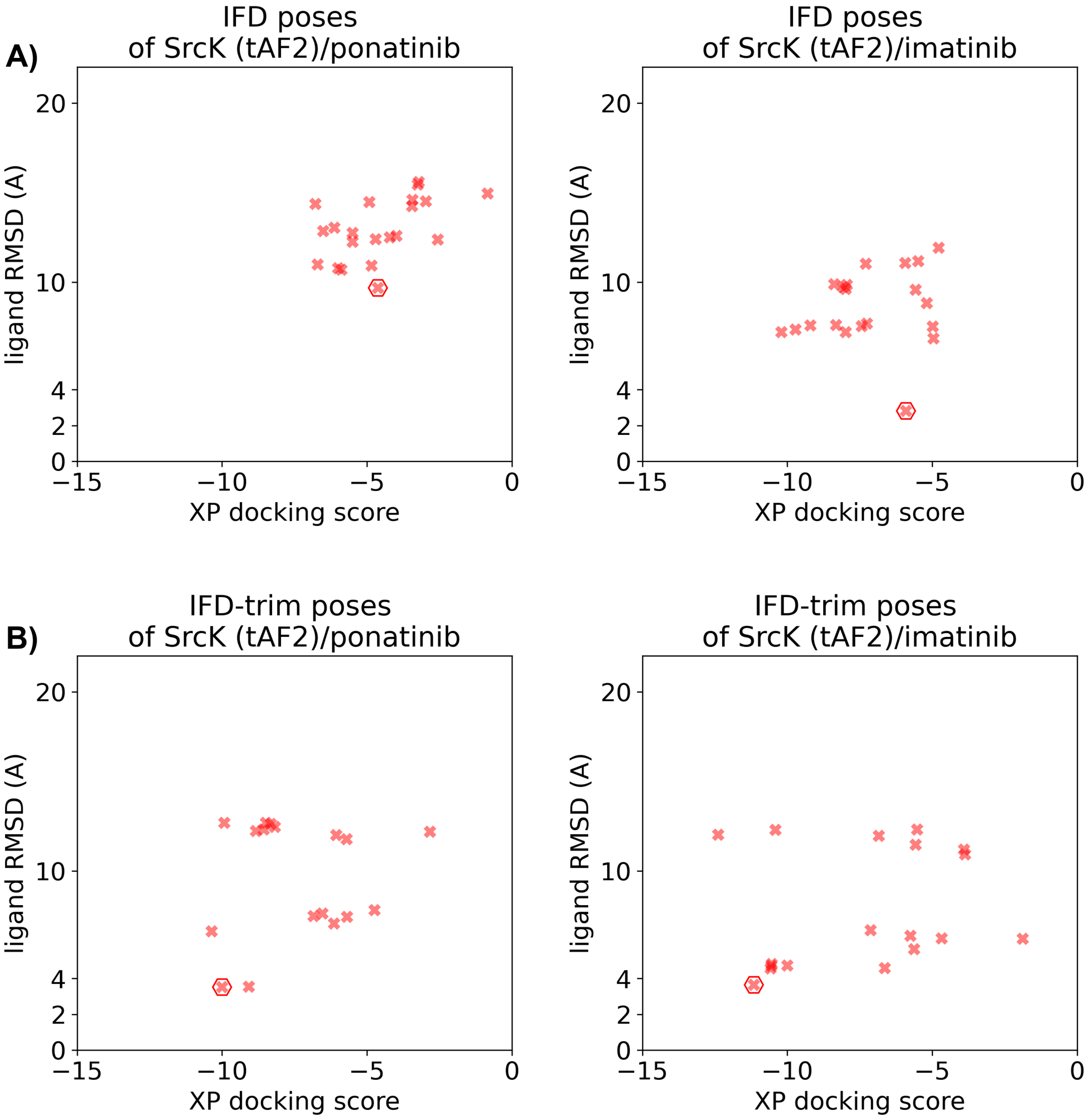

Figure S19: Ligand RMSDs are plotted against the docking scores for the IFD/IFD-trim docking poses of type II inhibitors (ponatinib and imatinib) against the SrcK tAF2 structure. The pose with the lowest ligand RMSD from each input structure is marked by hexagon.

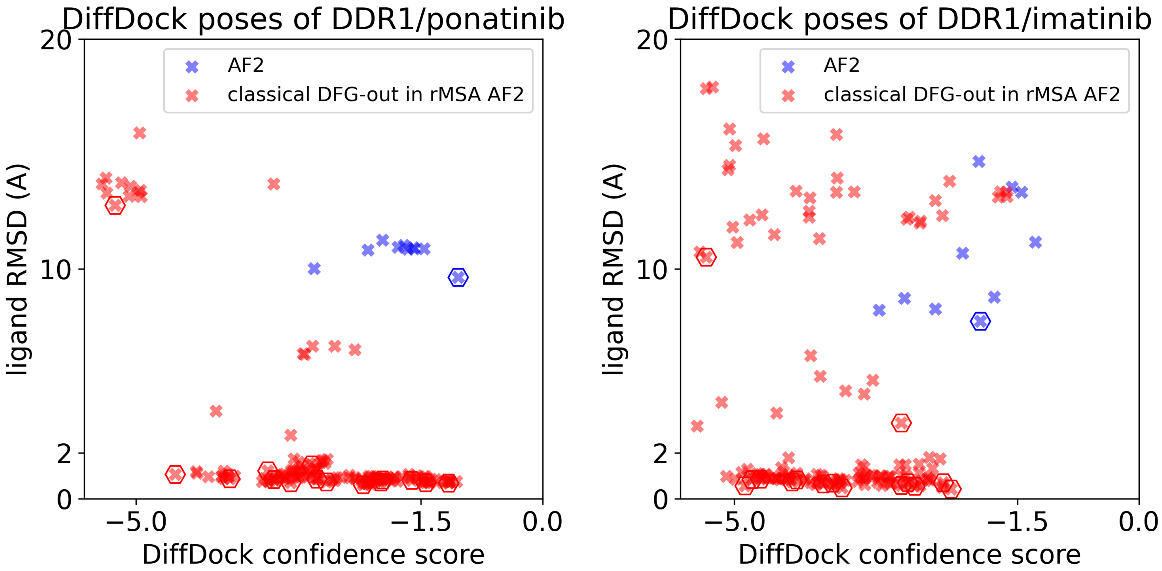

Figure S20: Ligand RMSDs are plotted against the DiffDock confidence scores for the DiffDock poses of type II inhibitors (ponatinib and imatinib) against DDR1 AF2 structure (blue) or the classical DFG-out structures in DDR1 rMSA AF2 ensemble (red). The pose with the lowest ligand RMSD from each input structure is marked by hexagon.

Table S1: Confidence score for the DiffDock pose aligns with AF2RAVE pmf values. The DiffDock confidence score of the pose with the lowest ligand RMSD (marked in red/bold) from each classical DFG-out structure in DDR1 rMSA AF2 ensemble is compared with the AF2RAVE pmf value for corresponding strutcure (marked in red/bold).

AF2RAVE pmf (kJ/mol)	Lowest ponatinib ligand RMSD (Å)	DiffDock conf_score	Lowest imatinib ligand RMSD (Å)	DiffDock conf_score
**0.37**	0.71	**-1.44**	0.55	-2.77
**0.57**	0.70	**-1.16**	0.43	-2.32
1.13	1.48	-2.84	3.31	-2.94
1.45	0.88	-3.85		-1000
1.62	0.82	-1.61	0.70	-2.42
2.07		-1000		-1000
2.69	0.62	-2.21	0.59	-2.94
3.82	0.86	-2.78	0.83	-4.23
4.02	0.71	-3.1	0.75	-4.29
4.21	0.82	-1.97	0.70	-2.86
4.39	12.77	-5.26	10.52	-5.35
4.70	0.85	-3.31	0.66	-3.9
7.60	1.22	-3.38	0.86	-4.68
N/A	0.75	-1.99	0.49	-3.67
N/A	0.75	-2.66	0.69	-3.79

References

(1) Hanson, S. M.; Georghiou, G.; Thakur, M. K.; Miller, W. T.; Rest, J. S.; Chodera, J. D.; Seeliger, M. A. Cell chemical biology **2019**, 26, 390–399.

(2) Vani, B. P.; Aranganathan, A.; Tiwary, P. Journal of Chemical Information and Modeling **2023**,

(3) Mirdita, M.; Schütze, K.; Moriwaki, Y.; Heo, L.; Ovchinnikov, S.; Steinegger, M. Nature methods **2022**, 19, 679–682.
