# Supplementary material for: Empowering AlphaFold2 for protein conformation selective drug discovery with AlphaFold2-RAVE
Source: ArXiv. 2024 May 12:arXiv:2404.07102v2. Originally published 2024 Apr 10. Preprint. [Version 2] (PMC11042445)
Supplement: Supplementary file 1 [file NIHPP2404.07102v2-supplement-1.pdf]

arXiv:2404.07102v2 [physics.bio-ph] 12 May 2024

Supplementary Information: Empowering AlphaFold2 for protein conformation selective drug discovery with AlphaFold2-RAVE

Xinyu Gu,[†] Akashnathan Aranganathan,[†,¶] and Pratyush Tiwary[*,†,‡,§]

†*Institute for Physical Science and Technology, University of Maryland, College Park, Maryland 20742, USA*

‡*Department of Chemistry and Biochemistry, University of Maryland, College Park 20742, USA*

¶*Biophysics Program, University of Maryland, College Park 20742, USA*

§*University of Maryland Institute for Health Computing, Bethesda, United States*

E-mail: ptiwary@umd.edu

rMSA AF2 ensemble generation

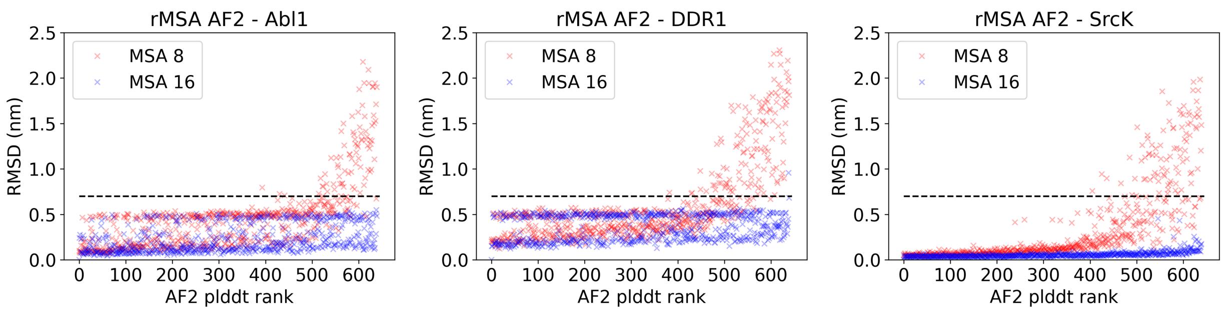

Figure S1: The AF2 pLDDT rank is plotted against the CA RMSDs from the AF2 structure (the one with the highest pLDDT) for each structure in the rMSA AF2 ensemble for Abl1, DDR1 or Src kinase. A RMSD cutoff of 7 Å (dashed black line) is applied to filter out unphysical structures with large RMSD from the native structure. Each rMSA AF2 ensemble is consist of 1280 structures, 640 for MSAs of depth 8:16 (red) and 16:32 (blue), separately.

AF2RAVE protocol on DDR1

Regular space clustering on the rMSA AF2 ensemble

We used the same 14 collective variables (CVs) for regular space clustering as in the previous AF2RAVE work on kinases.[1] These CVs are pariwise distances, selected to describe the kinase conformations around the ATP-binding pocket and the A-loop.

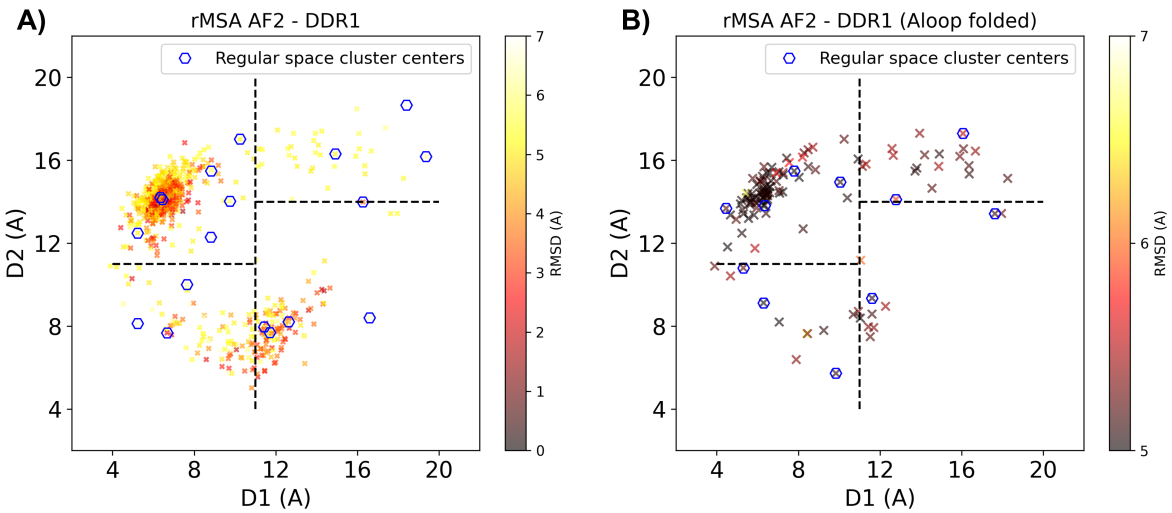

Figure S2: A) the rMSA AF2 ensemble for DDR1 is projected in the Dunbrack space. Sample points are color-coded based on the CA RMSD from the AF2 structure with the highest pLDDT. Regular space cluster centers are marked by blue hexagons. For each DFG-type (in, inter or out), top two cluster centers with the lowest CA RMSD are selected as AF2RAVE initial structures. B) To take account of the underrepresented A-loop folded configurations, an extra regular space clustering is conducted only for the A-loop folded structures in the rMSA AF2 ensemble. The color code, notation and the way to select initial structures are the same as plot A. Combining AF2RAVE initial structures from both plot A&B, there are 12 initial structures in total.

Unbiased MD and SPIB

50 ns unbiased MD simulation was run for each AF2RAVE initial structure of DDR1 from Fig S2. All the systems in this paper were parameterized with the Amber99SB*-ILDN force field[2] with the TIP3P water model.[3] The standard deviations of the 14 CVs are calculated after concatenating all the 12 unbiased MD trajectories. 8 CVs with standard deviations larger than 0.25 of the maximum standard deviation remain as the input features of the SPIB model. We conducted a parameter screening of the SPIB time lag, ranging from 1 ns to 40 ns with intervals of 1 ns. Eventually, we selected a time lag of 16 ns based on the performance of SPIB coordinates in representing physical features, including DFG-type and A-loop position.

PMF calculations from umbrella sampling

2D umbrella sampling is conducted along the two learnet SPIB coordinates, employing 11x11 windows. The bias potential equilibrium points are uniformly distributed in the SPIB latent space, with σ_1 ranging from -0.8 to -0.1 and σ_2 ranging from 0 to 0.8. The strength of the bias potential is set to 1000 $kJ/mol/nm^2$. Each window originates from the structure closest to the window's equilibrium point in Euclidean distance within the latent space among the 12 AF2RAVE initial structures and lasts for 100 ns (with the first 10 ns discarded in PMF calculation). For Abl1 kinase, we noticed a substantial proportion of the αC helix breaking in the umbrella sampling trajectories (Fig S9). This finding aligns with earlier enhanced sampling investigations on DDR1 using metadynamics,[1] where the authors imposed restraints to prevent αC helix breakage. In our study, we opted to exclude all umbrella sampling windows where the ratio of broken αC helix exceeded 20% for the Abl1 PMF calculation (Fig S6). The WHAM algorithm is applied to reweight the biased trajectories and compute the final PMF.

We must acknowledge the limitations of the way we assigned PMF values to AF-generated candidate holo structures. First of all, the free energy profiles are derived from MD simulations, and the PMF values directly correspond to the MD structures. We assumed that the latent space adequately represents the conformational changes of protein pocket within specific metastable states. Therefore, we project AF2-generated structures into the latent space and directly assign PMF values of the MD structures to AF-generated structures if they fall into the same bins in the latent space. Additionally, while the enrichment of holo structures in the top Boltzmann-ranked structures persists, the absolute PMF values and Boltzmann ranks of proper holo structures may fluctuate with the umbrella sampling setups, as depicted in Fig S7. Theoretically, the number of umbrella sampling windows and the simulation length should be sufficiently large for PMF convergence. However, there is always a trade-off between PMF accuracy and computational costs, so we opted to stick with the current setups.

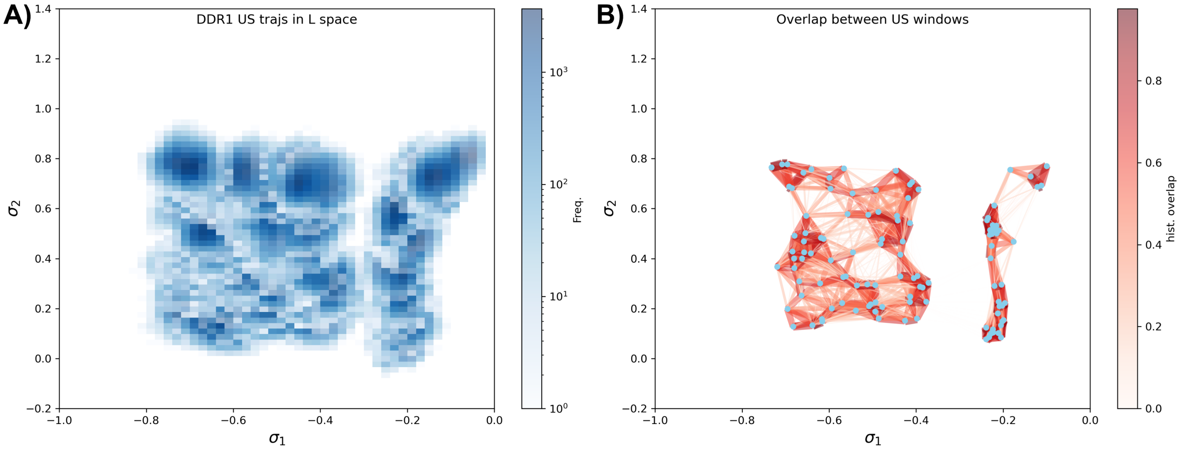

Figure S3: A) Distributions from different umbrella sampling windows in the latent space. B) The distribution overlap graph for all the umbrella sampling windows. The mean value of each distribution is shown as blue dots. Each distribution's 2D histogram is flattened into 1D vectors, and the cosine similarity between two distributions is then indicated by the width and color of the edge connecting the respective dots. Windows from the A-loop folded region are not overlapped well with the windows from the A-loop extended region, while windows inside the A-loop folded region (the left part of the graph) are well connected and are used for the local PMF calculation in Figure 4D.

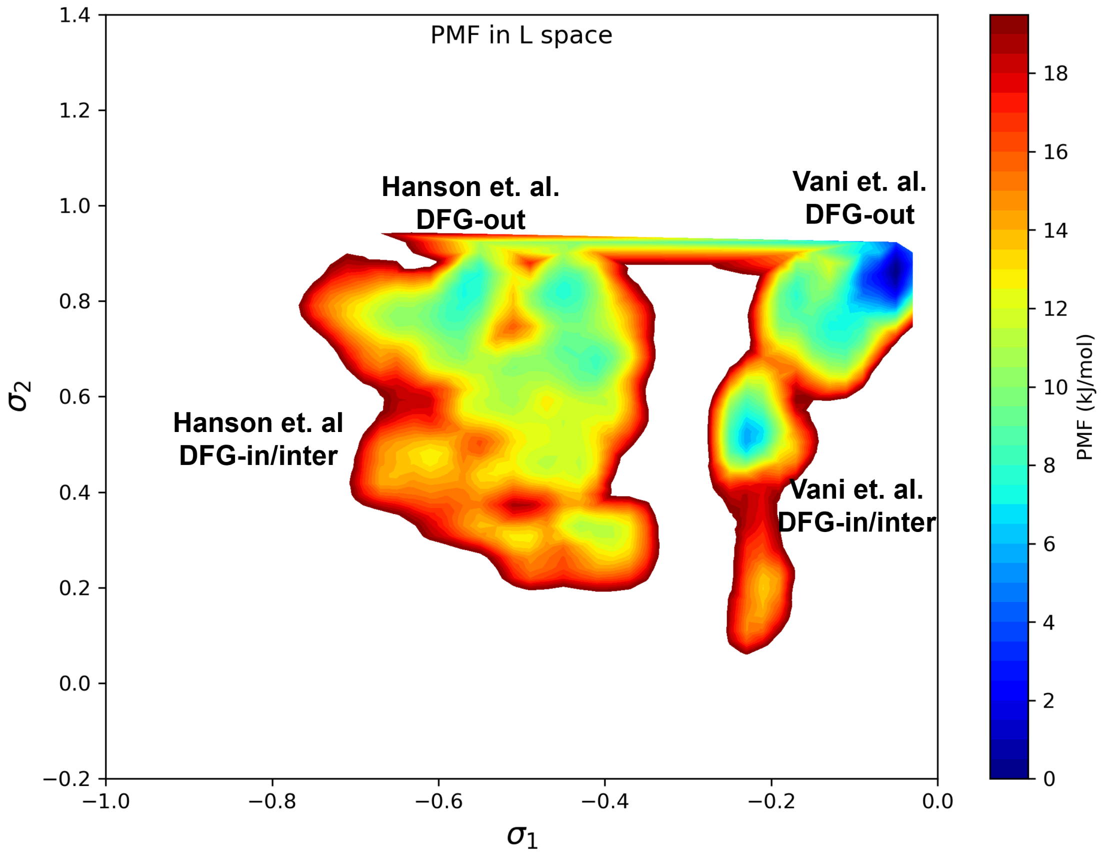

Figure S4: DDR1 PMF calculated with all the umbrella sampling windows. Hanson et al.[4] found the A-loop folded DFG-out state to be more stable than the A-loop folded DFG-in/inter state for DDR1; Vani et al.[1] reported that the A-loop extended DFG-out state is more stable than the A-loop extended DFG-in/inter state for DDR1. Although our umbrella sampling setup is not sufficient to sample the A-loop movement, the observed relative stability corresponds with the findings of Hanson et al. and Vani et al.

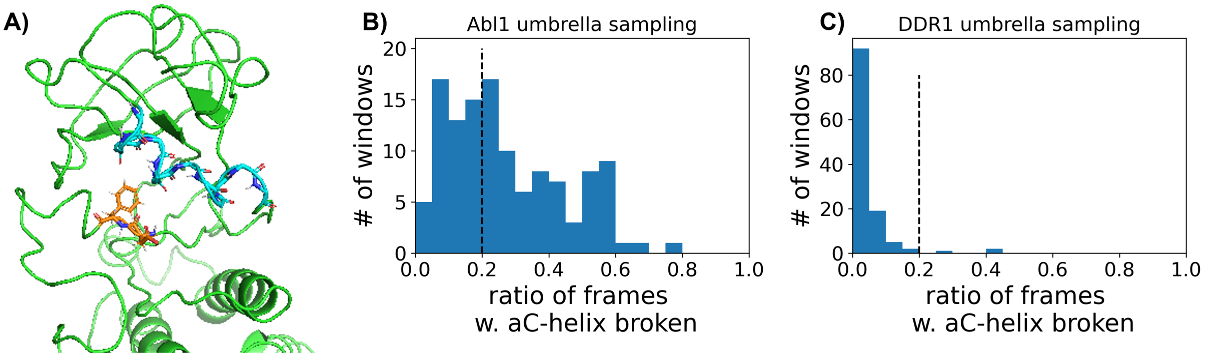

Figure S5: A) one representative frame with αC helix broken in Abl1 umbrella sampling trajectories. The backbone of the αC helix is shown with cyan sticks, while the DFG motif is shown as orange sticks. B) or C) The distribution of the ratios of frames with αC helix broken in each umbrella sampling window for Abl1 or DDR1.

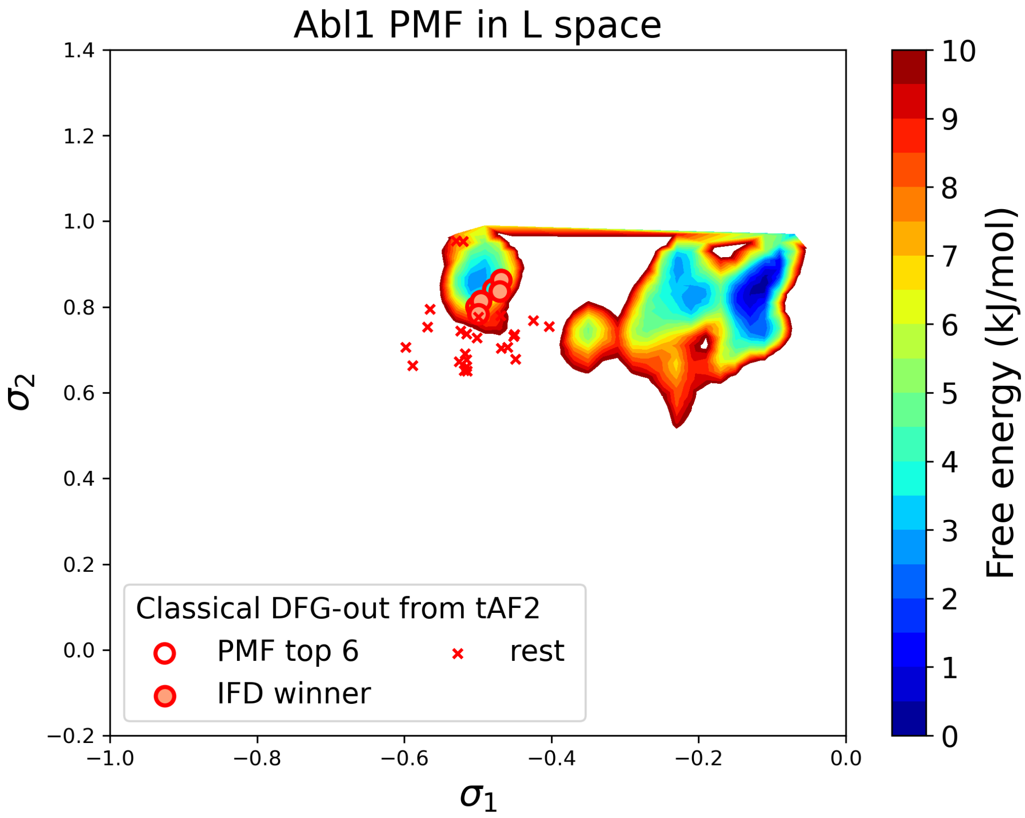

Figure S6: Abl1 PMF calculated from umbrella sampling after discarding windows with αC helix broken. The four holo-like structures (IFD winners) are enriched to the top six based on PMF values.

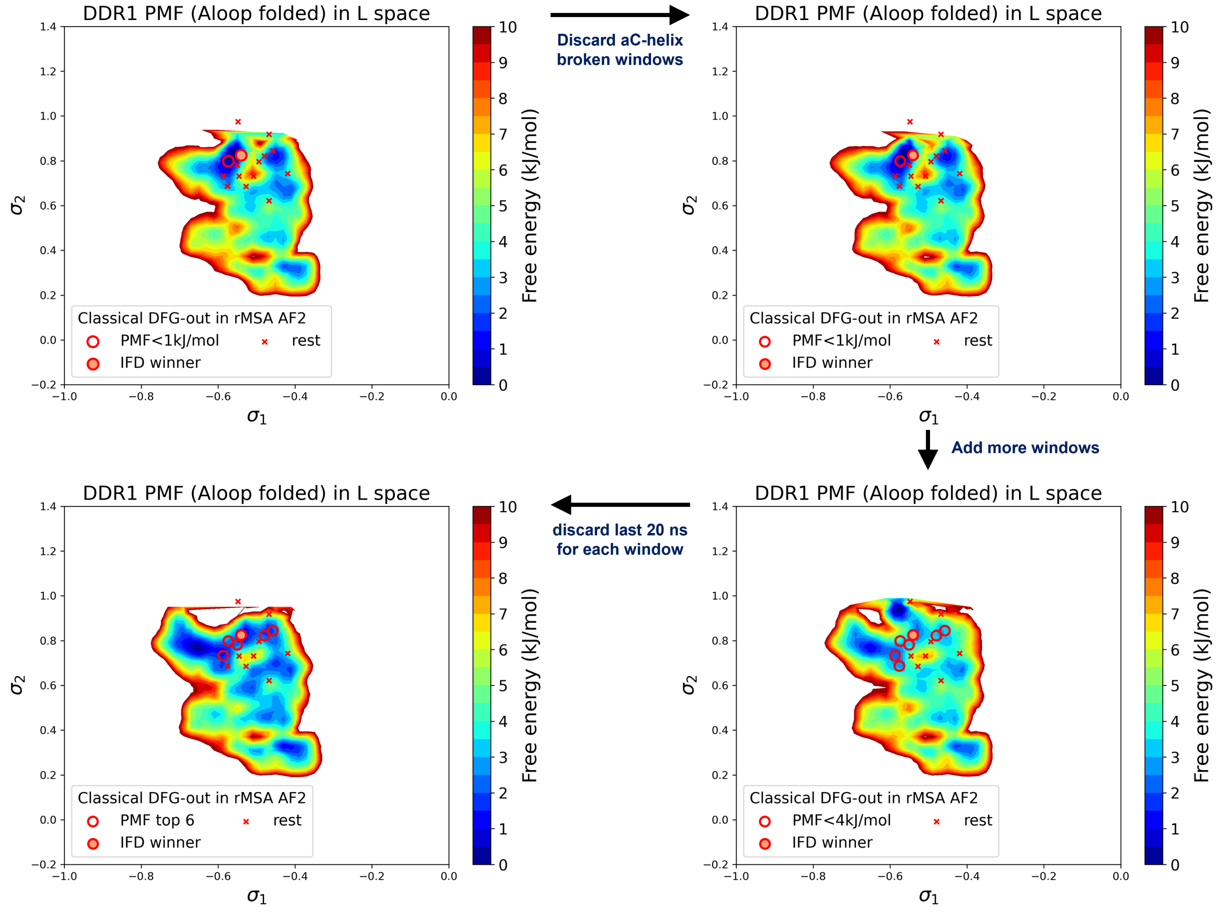

Figure S7: PMF values and Boltzmann ranks of candidate structures fluctuate with the selection of the umbrella sampling windows and the simulation length of umbrella sampling trajectories, demonstrated with the DDR1 system.

AF2-template

The AF2-based homology modeling protocol, AF2-template is implemented by uploading desired template structure to the colabfold[5] and deactivating the evoformer module. For each template, 5 AF2-template models are generated, with the last 3 structures exhibiting low pLDDT discarded.

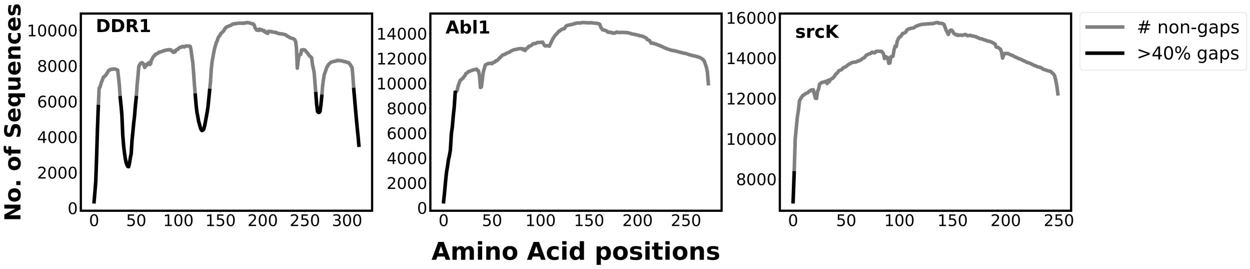

Figure S8: The plot illustrates the number of gaps in the multiple sequence alignment (MSA) generated by mmseq2 (using Colabfold[5]) for different kinases. The non-gap count describes the coverage of each position in the MSA. The presence of residue positions with gap counts higher than 40 per cent of the total sequence in DDR1 implies that it has fewer conserved regions than abl1 kinase and src kinase. This characteristic of DDR1 MSA enables the rMSA AF2 protocol to generate multiple conformations for DDR1, including the classical DFG-out conformation, by initializing it at various states. However, the highly conserved nature of abl1 and src makes it challenging for the rMSA AF2 to initialize at a state that can lead to a classical DFGout conformation. Therefore, we used the AlphaFold template protocol to overcome this initialization issue with rMSA AF2.

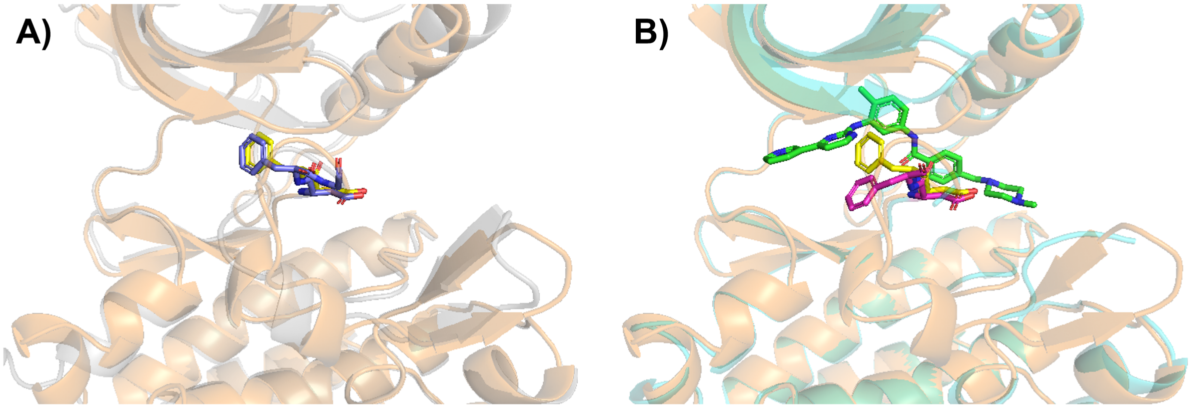

Figure S9: A) the AF2-template structure for Src kinase is superimposed with its template structure (classical DFG-out in DDR1 rMSA AF2 ensemble, IFD winner). The tAF2 structure of Src is shown as light-orange cartoon (protein) and yellow sticks (DFG motif), while DDR1 template is shown as light-gray cartoon (protein) and blue sticks (DFG motif). B) the AF2-template structure for Src kinase is again superimposed with Src/imatinib co-crystallized structure (PDB 2OIQ). Crystal structure is shown as light-cyan cartoon (protein), green sticks (ligand) and magenta sticks (DFG motif).

PMF calculations from unbiased MD

50 ns unbiased MD simulation was run starting from each structure in the 15 classical DFG-out structures in DDR1 rMSA AF2 ensemble. Upon discarding first 10 ns, all the unbiased trajectories are simply concatenated to calculate the Boltzmann distribution and PMF around the classical DFG-out basin. For Abl1, unbiased simulations start from 30 AF2-template structures to calculate the PMF around the classical DFG-out basin.

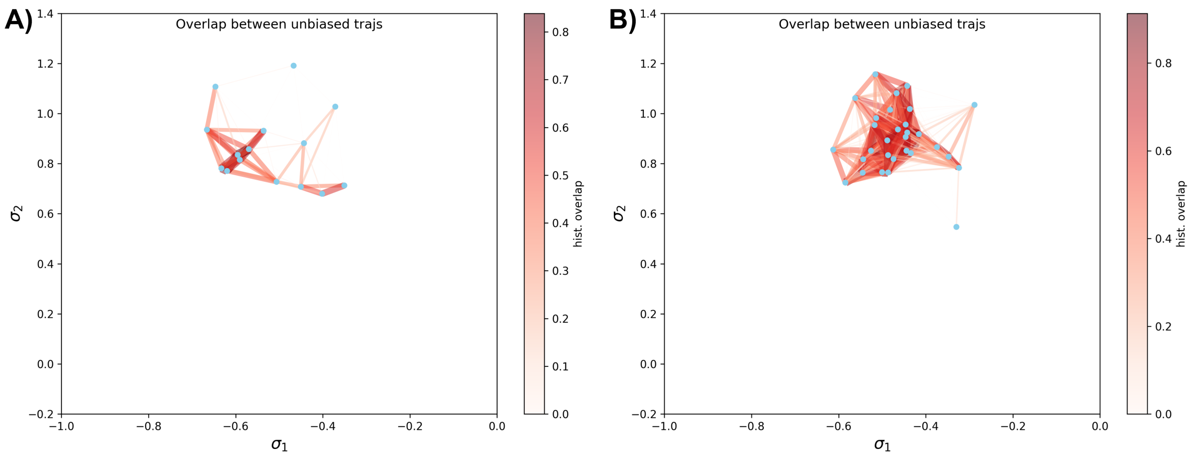

Figure S10: A) The distribution overlap graph for all the unbiased MD trajectories starting from 15 classical DFG-out structures in DDR1 rMSA AF2 ensemble. B) The distribution overlap graph for all the unbiased MD trajectories starting from 30 Abl1 tAF2 structures in classical DFG-out state. The color-code is the same as Figure S3

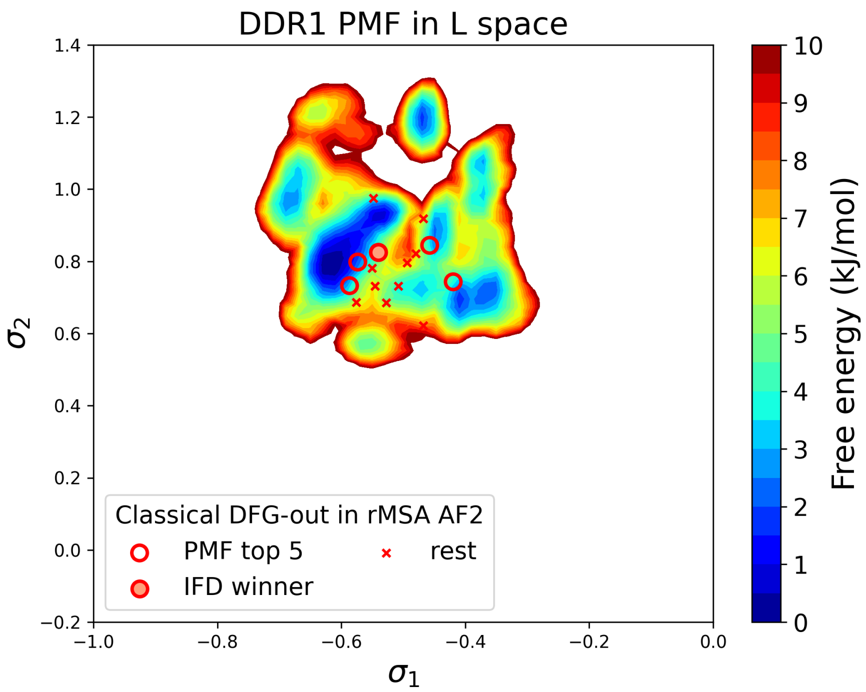

Figure S11: Free energy profile for DDR1 in the latent space, calculated from unbiased MD simulations. The 15 DDR1 classical DFG-out structures in rMSA AF2 are shown as red cross and circles (top 5 structures ranked by free energy values). The IFD winner structure is emphasized using a red circle filled with red.

AF2-cluster

AF2-cluster for DDR1 and Abl1 were run with default setups.[6]

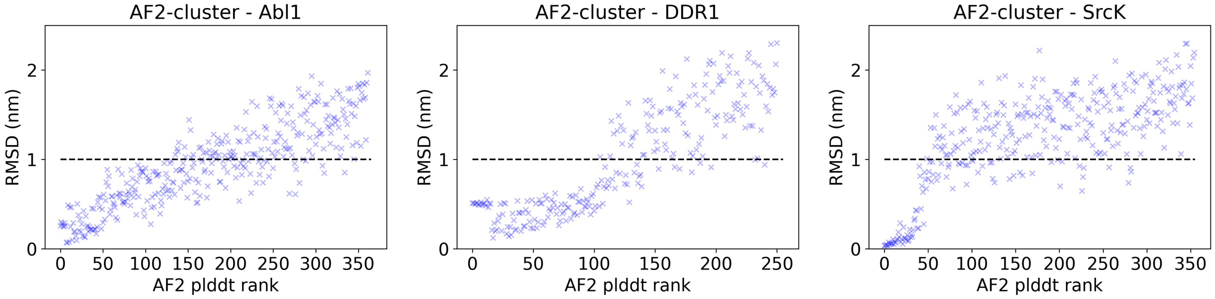

Figure S12: The AF2 pLDDT rank is plotted against the CA RMSDs from the AF2 structure for each structure in the AF2-cluster ensemble for Abl1, DDR1 or SrcK. A RMSD cutoff of 10 Å (dashed black line) is applied to filter out unphysical structures with large RMSD from the native structure. After the RMSD filter, 197 out of 362 structures remain for Abl1, 134 out of 251 structures remain for DDR1, and 93 out of 355 structures remains for SrcK.

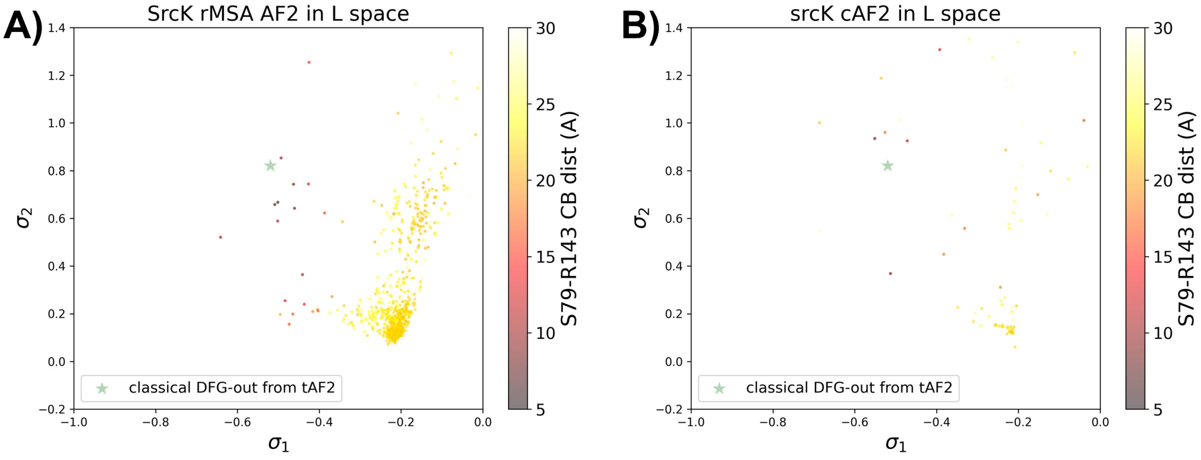

Figure S13: The projection of A) the rMSA AF2 ensemble or B) the AF2-cluster ensemble on the AF2RAVE latent space for SrcK. The classical DFG-out SrcK structure generated from AF2-template in Fig S9 is shown as the green star. The color-code shows the A-loop location.

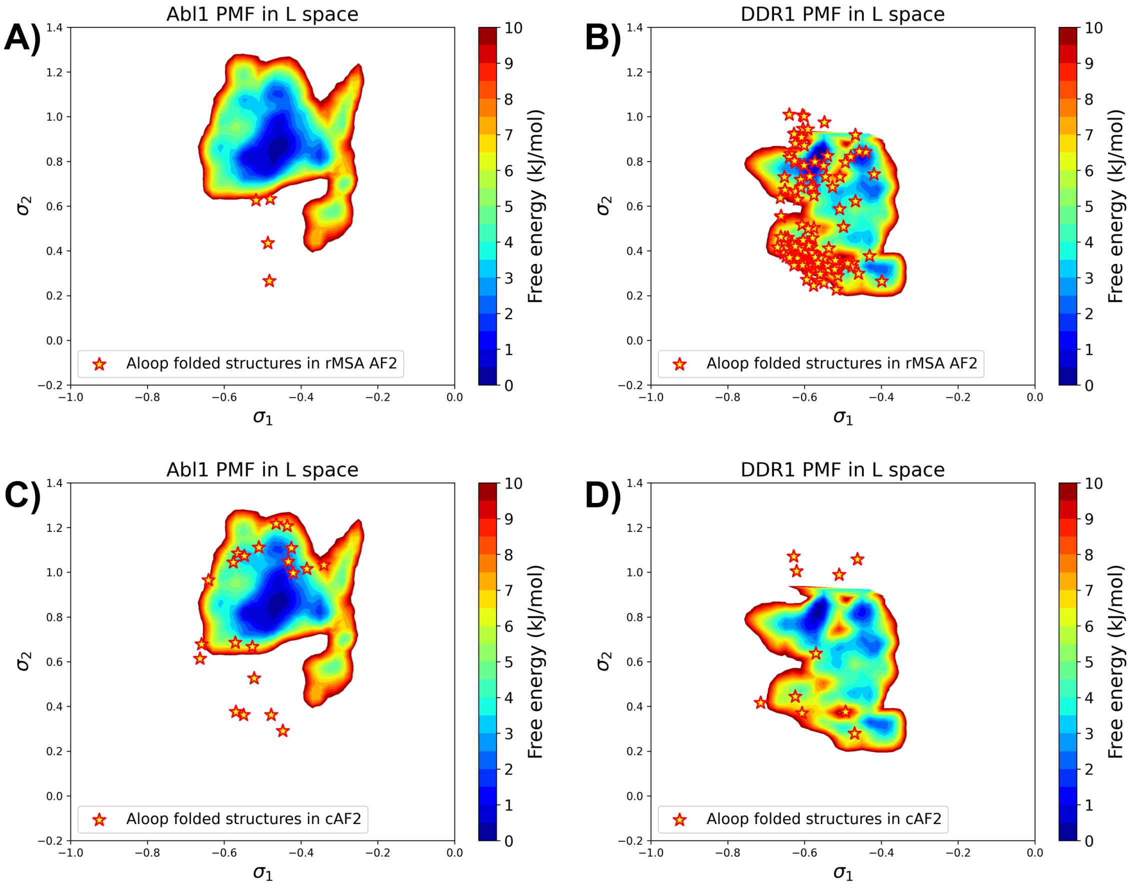

Figure S14: The projection of A-loop folded structures from the rMSA AF2 ensemble or the AF2-cluster ensemble on the AF2RAVE PMF for Abl1 or DDR1.

Docking details

All the input structure for our docking experiments were relaxed in solution first with a MD energy minimization step.

Glide XP

Glide XP docking experiments in this work were run with default setups in the Maestro software.

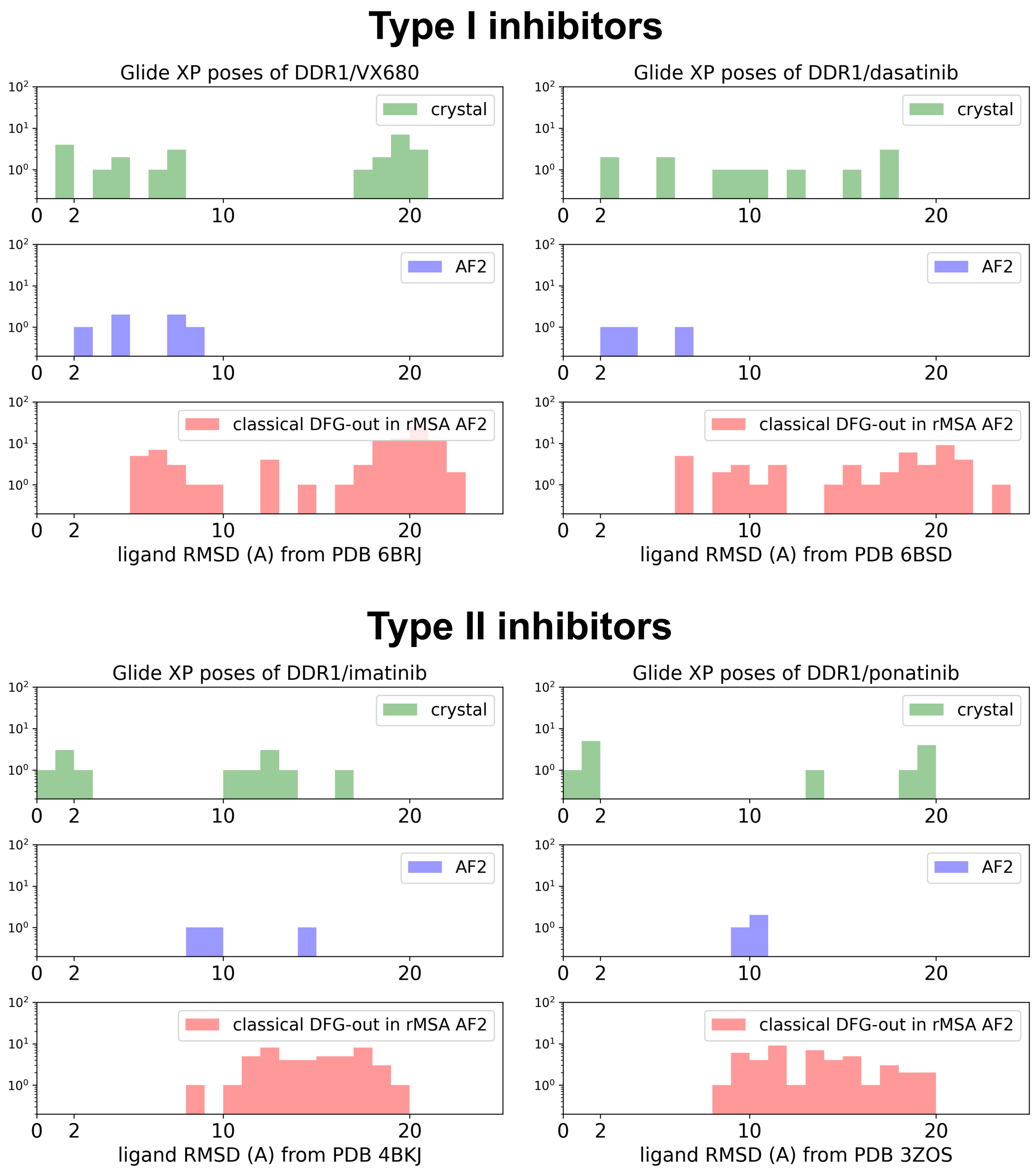

Figure S15: The distributions of ligand RMSDs for Glide XP docking poses of DDR1 and type I/type II inhibitors (upper/lower panel). Results from cross-docking against 4 crystal holo structures, docking against the AF structure, and docking against 15 classical DFG-out structure in rMSA AF2 ensemble are shown as green, blue, and red, separately.

IFD

For Induced-fit Docking, we used the Glide XP for initial docking, followed by Prime relaxation and final Glide XP docking. Parameters remain default in Maestro IFD.

Given Ponatinib's backbone feaftures, notably its lengthy and slender C-C triple bond, it exhibits reduced sensitivity to steric clashes, resulting in successful docking with the DDR1 IFD winner structure at a ligand RMSD of 0.89 Å. Conversely, the IFD winner structure struggles to accurately dock with imatinib using IFD (Fig S16 B). We then employed an extended-sampling IFD approach by initially trimming the DFG-Phe residue from the IFD winner structure. The trimmed residue is temporarily mutated to alanine during initial docking and later restored in subsequent Prime relaxation and final docking steps. Here, in the IFD winner structure, we manually chose the DFG-Phe residue which is significantly hindered by holo-imatinib (Fig S17 B). For generic systems lacking groundtruth information, broader screening of single residue trimming for the protein pocket may be necessary for this extended-sampling IFD method. In essence, it's a trade-off between the quality of holo-like structure to dock with and the accuracy/complexity of the docking method.

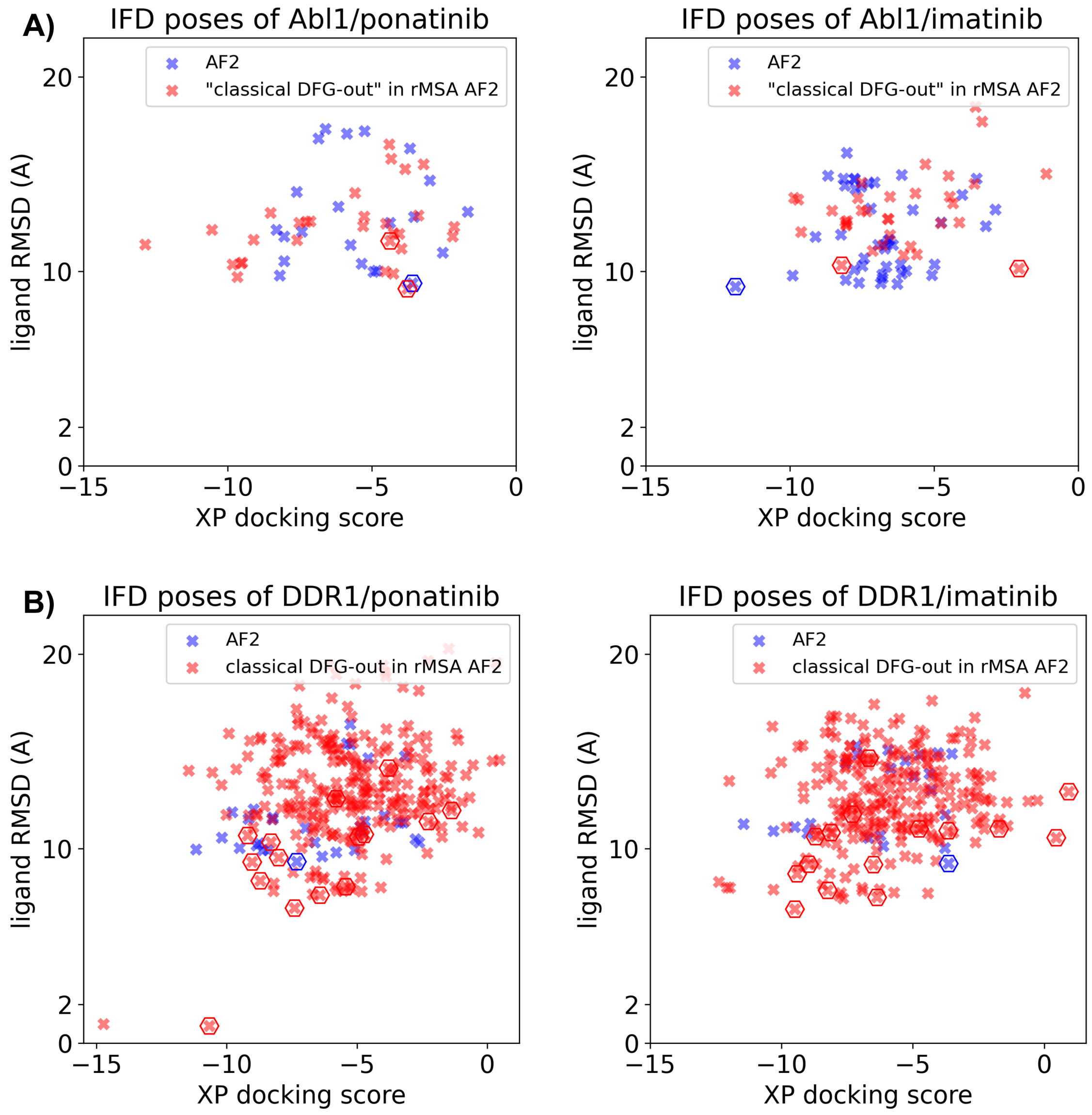

Figure S16: Ligand RMSDs are plotted against the docking scores for the IFD docking poses of type II inhibitors (ponatinib and imatinib) against AF2 structure (blue) or classical DFG-out structures in rMSA AF2 ensembles (red). A) IFD docking results for Abl1. B) IFD docking results for DDR1. The pose with the lowest ligand RMSD from each input structure is marked by hexagon.

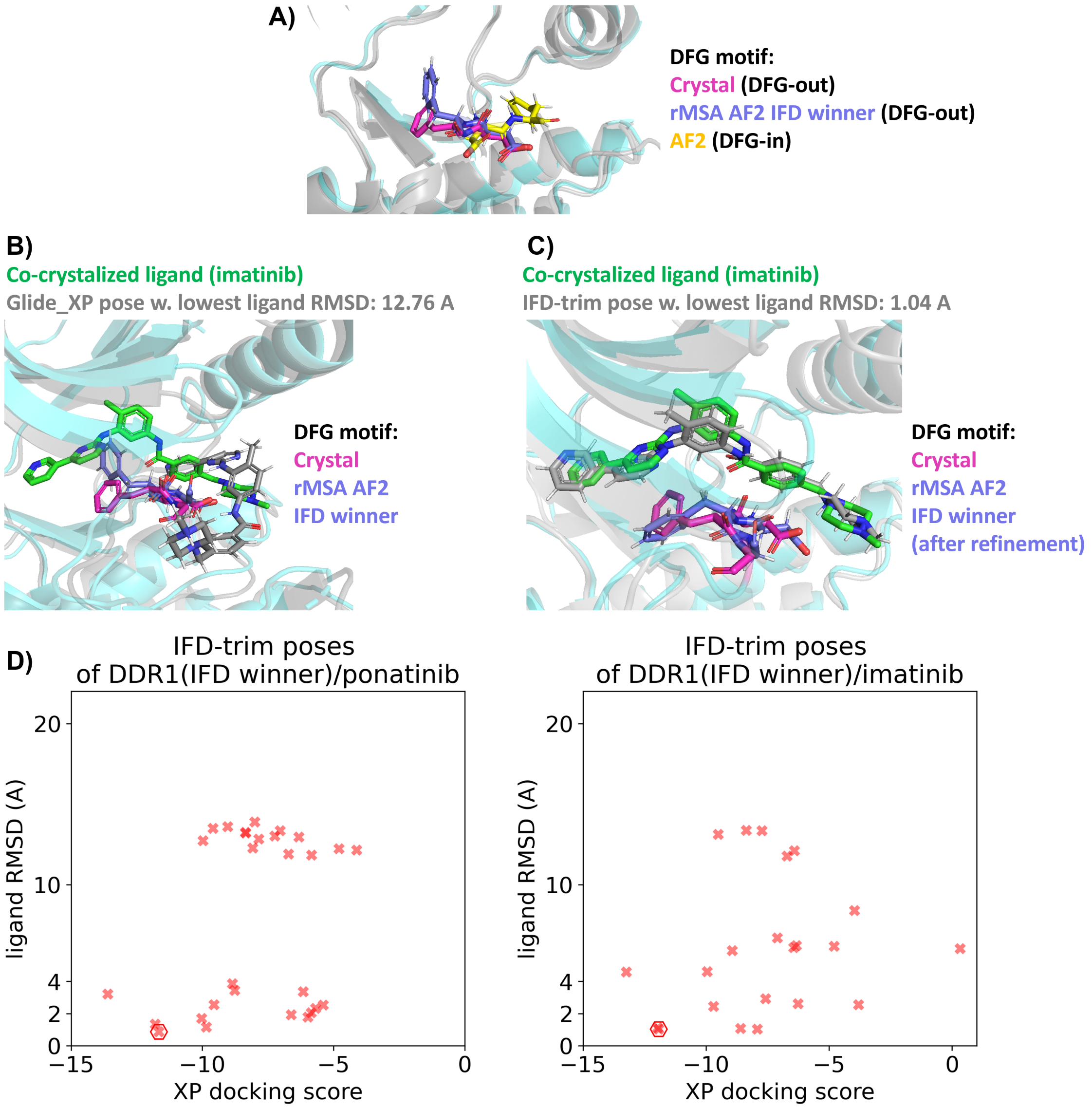

Figure S17: A) Comparison of the DFG motif for DDR1 in its co-crystalized structure with imatinib (PDB 4BKJ), its IFD winner structure and its AF2 structure. B&C) In the IFD winner structure, the Phe residue in the DFG-motif requires rotation to prevent steric clashes with imatinib. proteins from crystal structure are shown as cyan cartoon, while all the other proteins are shown as grey cartoon. D) Ligand RMSDs are plotted against the docking scores for the IFD-trim docking poses of type II inhibitors (ponatinib and imatinib) against the IFD winner structure in DDR1 rMSA AF2 ensembles. The pose with the lowest ligand RMSD is marked by hexagon.

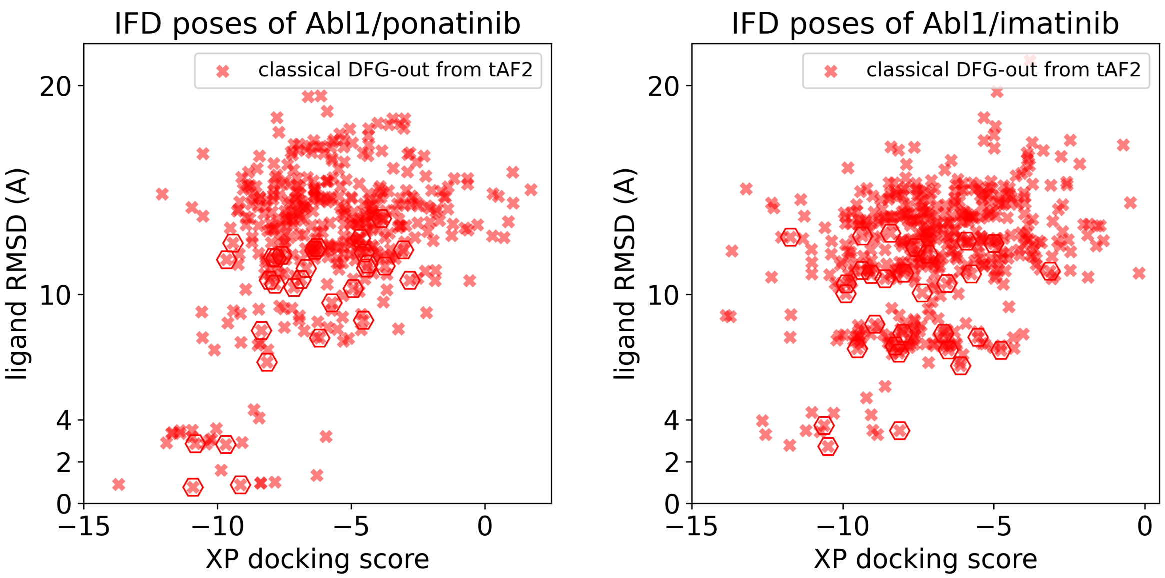

Figure S18: Ligand RMSDs are plotted against the docking scores for the IFD docking poses of type II inhibitors (ponatinib and imatinib) against Abl1 tAF2 structures. The pose with the lowest ligand RMSD from each input structure is marked by hexagon.

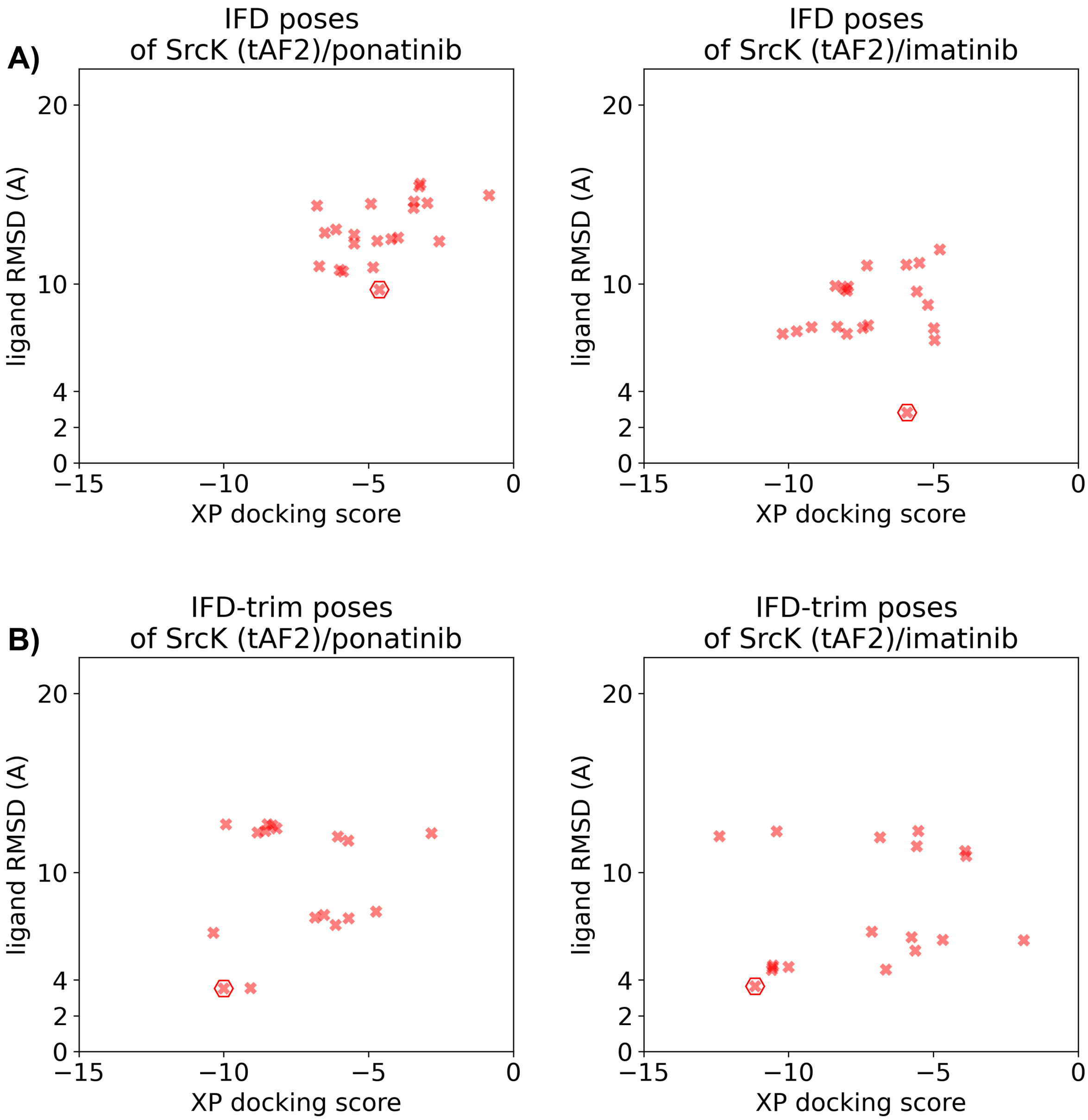

Figure S19: Ligand RMSDs are plotted against the docking scores for the IFD/IFD-trim docking poses of type II inhibitors (ponatinib and imatinib) against the SrcK tAF2 structure. The pose with the lowest ligand RMSD from each input structure is marked by hexagon.

Diffdock performace on classical DFG-out DDR1

DiffDock docking experiments in this work were run in the webserver with default setups in the version before 3/8/2024 (https://huggingface.co/spaces/simonduerr/diffdock).[7]

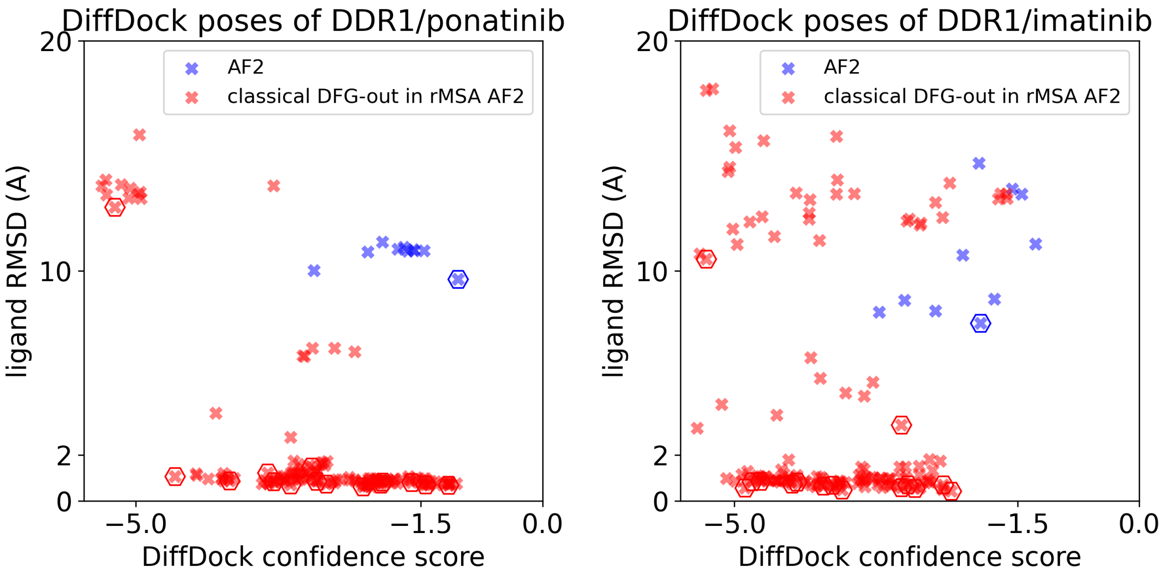

Figure S20: Ligand RMSDs are plotted against the DiffDock confidence scores for the DiffDock poses of type II inhibitors (ponatinib and imatinib) against DDR1 AF2 structure (blue) or the classical DFG-out structures in DDR1 rMSA AF2 ensemble (red). The pose with the lowest ligand RMSD from each input structure is marked by hexagon.

Table S1: Confidence score for the DiffDock pose aligns with AF2RAVE pmf values. The DiffDock confidence score of the pose with the lowest ligand RMSD (marked in red/bold) from each classical DFG-out structure in DDR1 rMSA AF2 ensemble is compared with the AF2RAVE pmf value for corresponding strutcure (marked in red/bold).

**AF2RAVE pmf (kJ/mol)**	**Lowest ponatinib ligand RMSD (Å)**	**DiffDock conf_score**	**Lowest imatinib ligand RMSD (Å)**	**DiffDock conf_score**
**0.37**	0.71	**-1.44**	0.55	-2.77
**0.57**	0.70	**-1.16**	0.43	-2.32
1.13	1.48	-2.84	3.31	-2.94
1.45	0.88	-3.85		-1000
1.62	0.82	-1.61	0.70	-2.42
2.07		-1000		-1000
2.69	0.62	-2.21	0.59	-2.94
3.82	0.86	-2.78	0.83	-4.23
4.02	0.71	-3.1	0.75	-4.29
4.21	0.82	-1.97	0.70	-2.86
4.39	12.77	-5.26	10.52	-5.35
4.70	0.85	-3.31	0.66	-3.9
7.60	1.22	-3.38	0.86	-4.68
N/A	0.75	-1.99	0.49	-3.67
N/A	0.75	-2.66	0.69	-3.79

References

(1) Vani, B. P.; Aranganathan, A.; Tiwary, P. Journal of Chemical Information and Modeling **2023**,

(2) Lindorff-Larsen, K.; Piana, S.; Palmo, K.; Maragakis, P.; Klepeis, J. L.; Dror, R. O.; Shaw, D. E. Proteins: Structure, Function, and Bioinformatics **2010**, 78, 1950–1958.

(3) Jorgensen, W. L.; Chandrasekhar, J.; Madura, J. D.; Impey, R. W.; Klein, M. L. The Journal of chemical physics **1983**, 79, 926–935.

(4) Hanson, S. M.; Georghiou, G.; Thakur, M. K.; Miller, W. T.; Rest, J. S.; Chodera, J. D.; Seeliger, M. A. Cell chemical biology **2019**, 26, 390–399.

(5) Mirdita, M.; Schütze, K.; Moriwaki, Y.; Heo, L.; Ovchinnikov, S.; Steinegger, M. Nature methods **2022**, 19, 679–682.

(6) Wayment-Steele, H. K.; Ojoawo, A.; Otten, R.; Apitz, J. M.; Pitsawong, W.; Hömberger, M.; Ovchinnikov, S.; Colwell, L.; Kern, D. Nature **2024**, 625, 832–839.

(7) Corso, G.; Stärk, H.; Jing, B.; Barzilay, R.; Jaakkola, T. arXiv preprint arXiv:2210.01776 **2022**,
